# [IPr^#^–PEPPSI]: A Well-Defined, Highly Hindered and Broadly Applicable Pd(II)–NHC (NHC = N-Heterocyclic Carbene) Precatalyst for Cross-Coupling Reactions

**DOI:** 10.3390/molecules28155833

**Published:** 2023-08-02

**Authors:** Md. Mahbubur Rahman, Qun Zhao, Guangrong Meng, Roger Lalancette, Roman Szostak, Michal Szostak

**Affiliations:** 1 Department of Chemistry, Rutgers University, 73 Warren Street, Newark, NJ 07102, USA; rahman.mm11@gmail.com (M.M.R.); qzhao23@jhu.edu (Q.Z.); gmeng@scripps.edu (G.M.); roger.lalancette@gmail.com (R.L.); 2 State Key Laboratory of Biocatalysis and Enzyme Engineering, School of Life Sciences, Hubei University, Wuhan 430000, China; 3 Department of Chemistry, Wroclaw University, F. Joliot-Curie 14, 50-383 Wroclaw, Poland; roman.szostak@chem.uni.wroc.pl

**Keywords:** [IPr^#^–PEPPSI], bulky yet flexible, palladium, air-stable catalysts, cross-coupling, chemoselective reactions

## Abstract

In this Special Issue, “Featured Papers in Organometallic Chemistry”, we report on the synthesis and characterization of [IPr^#^–PEPPSI], a new, well-defined, highly hindered Pd(II)–NHC precatalyst for cross-coupling reactions. This catalyst was commercialized in collaboration with MilliporeSigma, Burlington, ON, Canada (no. 925489) to provide academic and industrial researchers with broad access to reaction screening and optimization. The broad activity of [IPr^#^–PEPPSI] in cross-coupling reactions in a range of bond activations with C–N, C–O, C–Cl, C–Br, C–S and C–H cleavage is presented. A comprehensive evaluation of the steric and electronic properties is provided. Easy access to the [IPr^#^–PEPPSI] class of precatalysts based on modular pyridine ligands, together with the steric impact of the IPr^#^ peralkylation framework, will facilitate the implementation of well-defined, air- and moisture-stable Pd(II)–NHC precatalysts in chemistry research.

## 1. Introduction

Transition-metal-catalyzed cross-coupling reactions have been established as a transformative tool for organic synthesis in both academic and industrial research [1,2,3,4]. In particular, palladium-catalyzed cross-couplings have gained essential status in modern organic synthesis due to the versatility, broad functional group tolerance and predictability of the Pd(0)/(II) cycle under functional-group-tolerant and ligand-controlled reaction conditions [5,6,7,8,9,10,11,12,13,14,15,16]. At present, in addition to everyday applications in academic research, Pd-catalyzed cross-coupling reactions are implemented in ton-scale industrial processes and used by medicinal and materials chemists to develop more effective pharmaceuticals and new functional materials that are essential for quality of life [1,2,3,4,5,6,7,8,9,10,11,12,13,14,15,16].

Nucleophilic N-heterocyclic carbenes (NHCs) have been developed as a particularly attractive class of ligands for Pd-catalyzed cross-coupling reactions [17,18,19,20,21,22,23,24,25,26,27,28,29,30,31,32]. Primarily, the strong σ-donation and variable steric hindrance of the N-Ar wingtips of the NHC scaffold facilitate oxidative addition and the reductive elimination steps of the catalytic cycle and open up new directions for cross-coupling research [33,34,35,36,37]. Furthermore, Pd–NHC catalysts have also been employed in other classes of Pd-catalyzed transformations, including oxidative couplings [38], polymerizations [39] and radical couplings [40], where the well-defined topology of NHC ligands enables the specific control of the elementary steps and selectivity of the processes.

**Figure 1:**
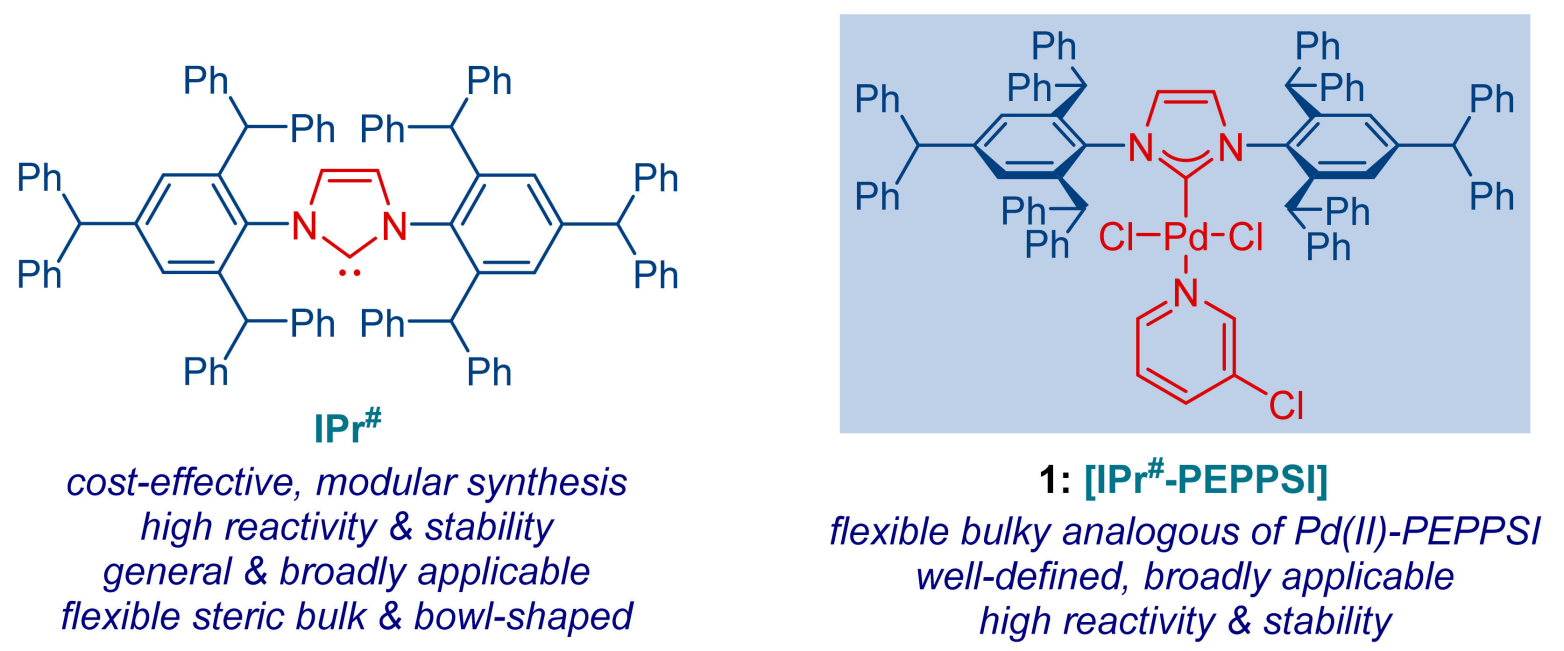
Sterically demanding IPr^#^ and [IPr^#^-PEPPSI].

We recently reported on a novel class of sterically hindered NHC ligands obtained through the modular peralkylation of aniline (Figure 1) [41,42]. These ligands combine the steric properties of sterically demanding NHCs with facile multigram-scale synthesis in a cost-effective manner utilizing feedstock aniline that is available in bulk. Furthermore, the para-substituent stabilizes the N-Ar group from rotation, enabling the improved steric control of the ortho-substituents in the catalytic pocket. Our initial studies were performed using allyl-based catalysts, such as [(IPr^#^)Pd(cin)Cl] (MilliporeSigma, no. 919616) [41,43]. Since it is well-established that Pd–PEPPSI complexes are complementary to Pd–allyl-based catalysts and should be screened together with Pd-allyl NHC complexes [44,45], we investigated [IPr^#^–PEPPSI] as a readily prepared Pd(II)–NHC complex.

In this Special Issue, “Featured Papers in Organometallic Chemistry”, we report on the synthesis and characterization of [IPr^#^–PEPPSI], a highly hindered Pd(II)–NHC precatalyst for cross-coupling reactions (Figure 1) [46]. More specifically, this complex features 3-Cl-py as an ancillary ligand, [(IPr^#^)Pd(3-Cl-py)Cl_2_] (**1**), and belongs to the PEPPSI-type class of catalysts. This catalyst was commercialized in collaboration with MilliporeSigma (no. 925489) to provide academic and industrial researchers with broad access to reaction screening and optimization. The broad activity of [IPr^#^–PEPPSI] in cross-coupling reactions, as well as a comprehensive evaluation of its steric and electronic properties, is presented [17]. Easy access to [IPr^#^–PEPPSI] based on modular pyridine ligands, together with the steric impact of the IPr^#^ peralkylation framework, will facilitate the implementation of well-defined Pd(II)–NHCs in chemistry research.

## 2. Results

**Scheme 1:**
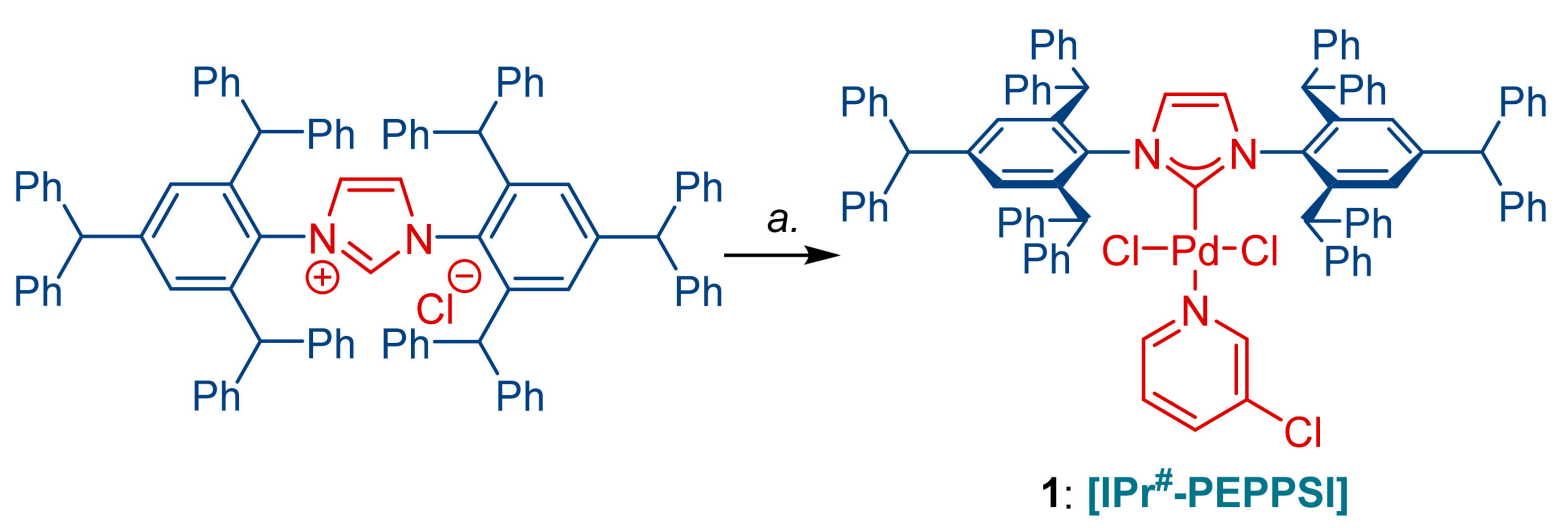
Synthesis of [IPr^#^–PEPPSI] catalyst. Conditions: IPr^#^·HCl (1.1 equiv), PdCl_2_ (1.0 equiv), K_2_CO_3_ (5.0 equiv), 3-Cl-py, 80 °C, 24 h, 82% yield.

Our studies commenced with the synthesis of the [IPr^#^–PEPPSI] precatalyst. As shown in Scheme 1, the reaction of IPr^#^HCl, PdCl_2_, K_2_CO_3_ and 3-Cl-py at 80 °C for 24 h afforded the desired complex, [IPr^#^–PEPPSI] (**1**), in an 82% yield [47,48]. Importantly, complex (**1**) was found to be stable in air and moisture.

**Figure 2:**
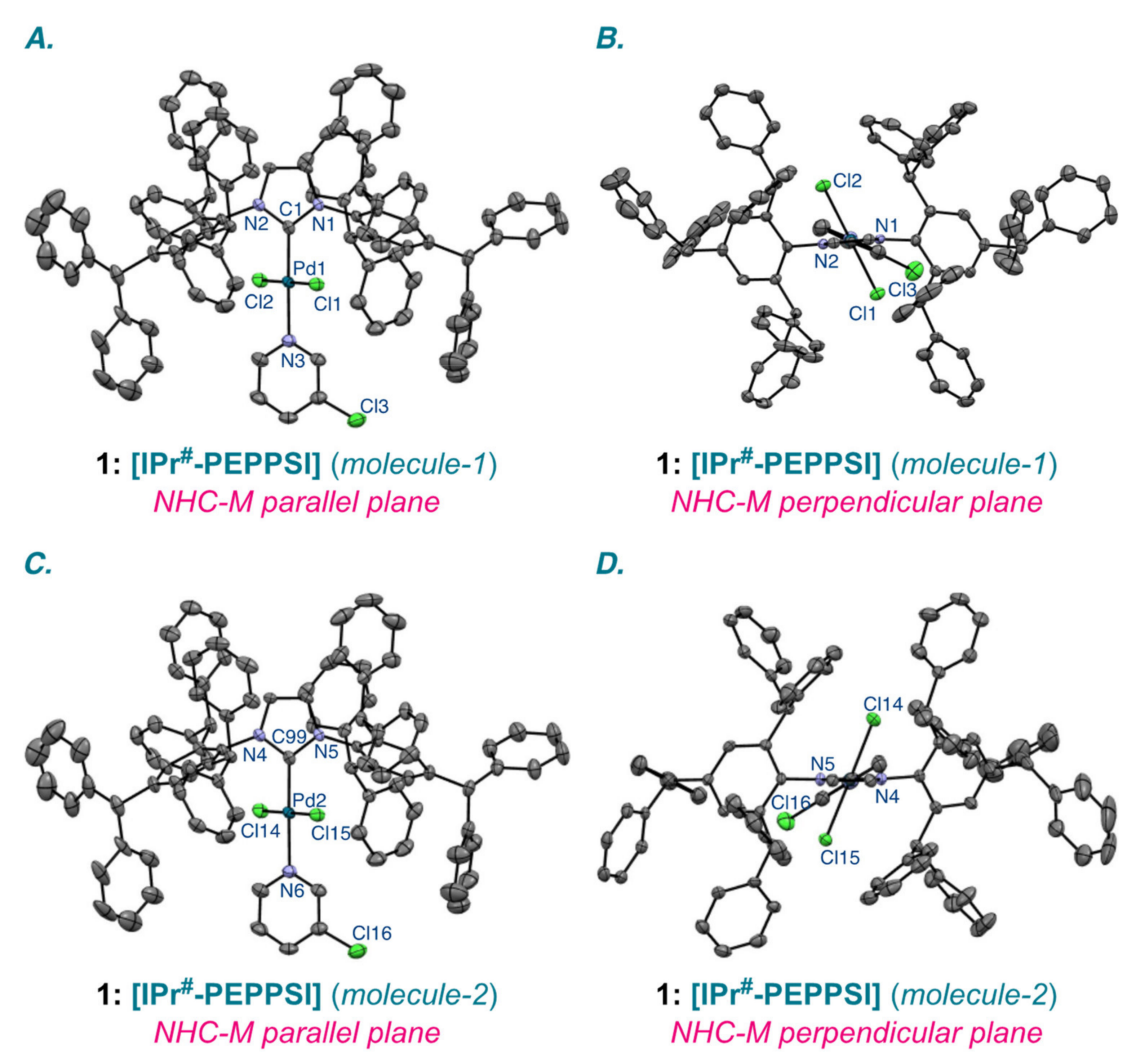
X-ray single-crystal structure of the complex [IPr^#^–PEPPSI] (**1**). Two molecules in the unit cell. (**A**) Molecule-1: NHC–M parallel plane; (**B**) molecule-1: NHC–M perpendicular plane; (**C**) molecule-2: NHC–M parallel plane; (**D**) molecule-2: NHC–M perpendicular plane; hydrogen atoms are omitted for clarity. Selected bond lengths (Å) and angles (°): molecule-1: Pd1–C1, 1.978(4); Pd1–Cl1, 2.285(1); Pd1–Cl2, 2.300(1); Pd1–N3, 2.119(4); C1–N1, 1.353(6); C1–N2, 1.356(6); C1–Pd1–Cl1, 88.8(1); C1–Pd1–Cl2, 91.5(1); N3–Pd1–Cl1, 90.6(1); N3–Pd1–Cl2, 89.1(1); molecule-2: Pd2–C99, 1.965(4); Pd2–Cl14, 2.301(1); Pd2–Cl15, 2.302(1); Pd2–N6, 2.114(4); C99–N4, 1.365(6); C99–N5, 1.354(6); C99–Pd2–Cl14, 89.8(1); C99–Pd2–Cl15, 89.4(1); N6–Pd2–Cl14, 90.4(1); N6–Pd2–Cl15, 90.4(1). CCDC 2262376.

The complex [IPr^#^–PEPPSI] (**1**) was fully characterized via x-ray crystallography (CCDC 2262376) (Figure 1). The single crystal was obtained through slow evaporation from dichloromethane from a dilute solution. The complex crystalized with two molecules in the unit cell. Similar to other Pd(II)-Het complexes, [IPr^#^–PEPPSI] (**1**) features a square planar geometry at the Pd center (Figure 2, Table 1) [47,48]. The bond angles between the ligands at the metal center in complex (**1**) (molecule-1: C–Pd–Cl, 88.8(1)°, 91.5(1)°; N–Pd–Cl, 90.6(1)°, 89.1(1)°; molecule-2: C–Pd–Cl, 89.8(1)°, 89.4(1)°; N–Pd–Cl, 90.4(1)°, 90.4(1)°) are consistent with the square planar geometry and in the range of Pd(II)–Het complexes bearing imidazole-2-ylidene ligands (e.g., [IPr–PEPPSI], C–Pd–Cl, 91.5(9)°, 87.2(9)°; N–Pd–Cl, 91.0(8)°, 90.4(7)°; [IPent–PEPPSI], C–Pd–Cl, 90.5(1)°, 90.3(1)°; N–Pd–Cl, 89.27(7)°, 89.90(7)°) [47,48]. The metal–ligand bond lengths of [IPr^#^–PEPPSI] (**1**) (molecule-1: Pd–C, 1.978(4) Å; Pd–Cl, 2.285(1) Å, 2.300(1) Å; Pd–N, 2.119(4) Å; molecule-2: Pd–C, 1.965(4) Å; Pd–Cl, 2.301(1) Å, 2.302(1) Å; Pd–N, 2.114(4) Å) can be compared with those of Pd(II)–Het complexes such as [IPr–PEPPSI] (Pd–C, 1.969(3) Å; Pd–Cl, 1.290(9) Å, 1.298(7) Å; Pd–N, 2.137(3) Å) and [IPent–PEPPSI] (Pd–C, 1.975(3) Å; Pd–Cl, 2.2868(9) Å, 2.3033(9) Å; Pd–N, 2.097(2) Å) [47,48].

It should be noted that the unit cell contains two molecules, and they are independent. For clarity, both of the molecules are discussed in the same manner. The atoms corresponding to the second molecule show larger ellipsoids than those of the first molecule, but the magnitude of the ellipsoids is very small. In the standard case, the anisotropic parameter for each atom is less than 0.2. There are two atoms (C198 and C174) for which the anisotropic parameter is slightly higher than 0.2. Despite our extensive attempts, the anisotropic parameter did not change.

**Figure 3:**
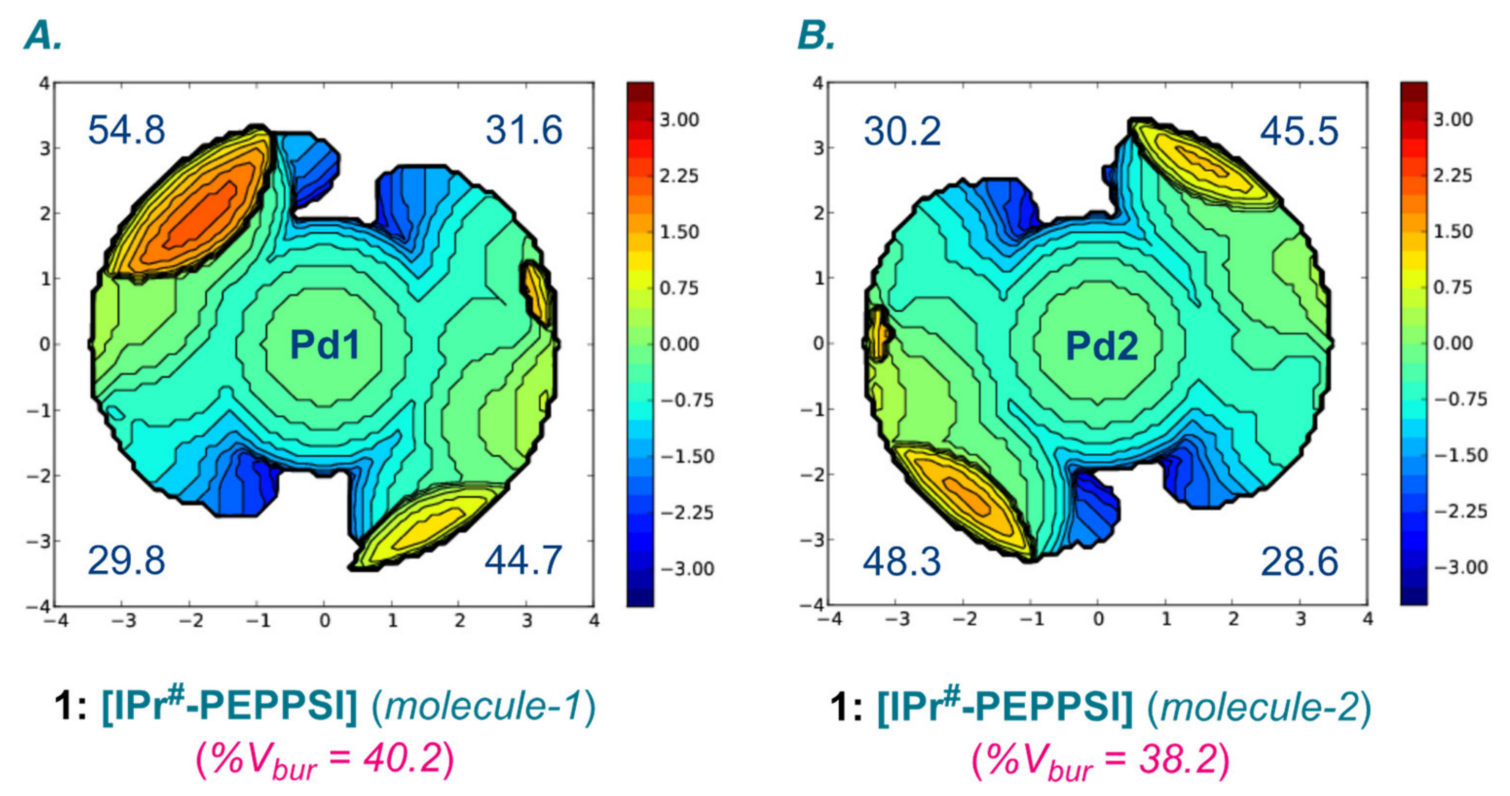
Topographical steric map of [IPr^#^–PEPPSI] (**1**) showing %V*_bur_* per quadrant calculated from the X-ray single-crystal structure. (**A**) molecule-1; (**B**) molecule-2. See Supplementary Materials for additional details.

To further evaluate the steric impact of the IPr^#^ ligand on the [IPr^#^-PEPPSI] (**1**) complex, the percent buried volumes (%V*_bur_*) were calculated using the method of Cavallo (Figure 3) [49,50]. The %V*_bur_* values of the IPr^#^ ligand in complex (**1**) are 40.2% (SW, 29.8%; NW, 54.8%; NE, 31.6%; SE, 44.7%) for molecule-1 and 38.2% (SW, 48.3%; NW, 30.2%; NE, 45.4%; SE, 28.6%) for molecule-2. These values can be compared with the %V*_bur_* of Pd–PEPPSI complexes, such as IPr (%V*_bur_*, 34.8%; SW, 32.7%; NW, 40.7%; NE, 29.1%; SE, 36.7%) and IPent (%V*_bur_*, 38.3%; SW, 47.4%%; NW, 30.3%%; NE, 45.2%; SE, 30.4%) [19]. Interestingly, the average steric impact of the IPr^#^ ligand in [IPr^#^–PEPPSI] (**1**) is higher than that in both the IPr and IPent congeners, although it is lower than that in the allyl-congener [Pd(IPr^#^)(cin)Cl] (%V*_bur_*, 44.7%; SW, 26.9%; NW, 63.7%; NE, 29.9%; SE, 58.2%) [41].

**Scheme 2:**
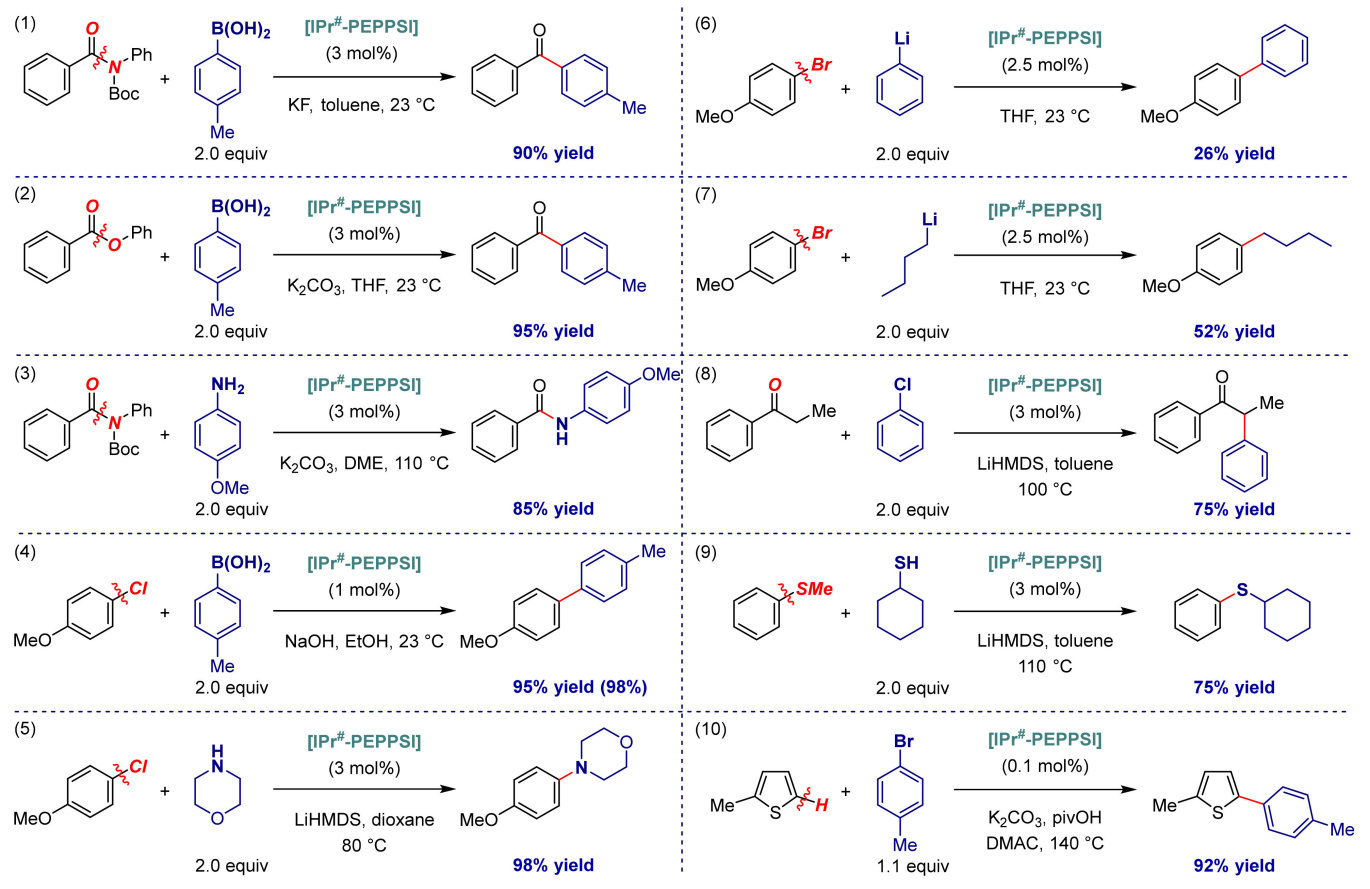
Activity of [IPr^#^–PEPPSI] in cross-coupling reactions.

Next, we evaluated the activity of [IPr^#^–PEPPSI] (**1**) in a range of cross-coupling reactions (Scheme 2) [41]. As shown, [IPr^#^–PEPPSI] (**1**) is a highly effective catalyst in acyl N–C(O) amide Suzuki cross-coupling (entry 1) [51,52], acyl O–C(O) ester Suzuki cross-coupling (entry 2) [53,54], the transamidation of acyl N–C(O) (entry 3) [55], C–Cl Suzuki (entry 4) [56] and C–Cl Buchwald–Hartwig cross-coupling (entry 5) [57], C–Br Murahashi–Feringa cross-coupling (entry 7) [58,59], ketone α-arylation (entry 8) [60], Morandi C–S sulfur cross-coupling (entry 9) [61] and C–H arylation (entry 10) [62]. It is now well-established that the selectivity of different Pd–NHCs depends on the stabilizing ancillary ligand, and it is generally recommended that Pd–NHCs with various ancillary ligands should be screened in order to identify the optimal catalyst for a given reaction. Importantly, the results demonstrate that [IPr^#^–PEPPSI] (**1**) is effective in a range of N–C, O–C, C–Cl, C–Br, C–S and C–H activations using different organometallic reagents (organoboron, amide, organolithium, enolate, sulfide) for cross-coupling reactions that are among the most common in industrial and academic settings [1,2,3,4,5,6,7,8,9,10,11,12,13,14,15,16]. Considering the availability of [IPr^#^–PEPPSI] (MilliporeSigma no. 925489) and its broad reactivity, this catalyst is an important addition to the cross-coupling toolbox [63,64].

It should be noted that steric flexibility is critical for stabilizing different intermediates in catalytic cycles. For example, it is well-known that less steric hindrance is better for OA and more steric hindrance is beneficial for RE [1,2,3,4,5,6,7,8,9]. The steric flexibility of the IPr# ligand confers the capacity to adjust to the required steric environment in the key catalytic steps. The asymmetric steric distribution likely contributes to the flexibility of the ligand [17,18,19,20,21,22,23,24]. Regarding the turnover-limiting steps, it is generally accepted that oxidative addition is the rate-limiting step for less reactive electrophiles, while reductive elimination is limiting for substrates that undergo facile oxidative addition [1,2,3,4,5,6,7,8,9]. To demonstrate the generality of the catalyst, the catalyst has been tested in a variety of different types of reactions, including electrophiles and nucleophiles that are characterized by a distinct mechanism.

In a broader sense, the present catalyst should be benchmarked against its closest analogue, [PdCl(IPr^#^)(cin)], which features the cinnamyl group instead of 3-chloro-pyridine as an ancillary ligand. The [PdCl(IPr^#^)(cin)] catalyst leads to the formation of the products in Scheme 2 in the following yields: 95% (entry 1), 40% (entry 2), 75% (entry 3), 98% (entry 4), 98% (entry 5), 96% (entry 6), 82% (entry 7), 90% (entry 8), 80% (entry 9) and 95% (entry 10). Thus, [IPr^#^–PEPPSI] is more effective than its allyl-congener, [(IPr^#^)Pd(cin)Cl], in O–C(O) cross-coupling (entry 2) but less effective in C–Br Murahashi–Feringa cross-coupling (entries 6–7), while its reactivity in other reactions is comparable (entries 1, 3–5, 8–10). In general, it is now well-established that the selectivity of different Pd–NHCs depends on the stabilizing ancillary ligand, and it is generally recommended that Pd–NHCs with various ancillary ligands should be screened in order to identify the optimal catalyst for a given reaction. It should be noted that the PEPPSI-type complexes are a distinct class of Pd-NHC catalysts, differing from (NHC)Pd(R-allyl)Cl and other classes of Pd-NHC complexes, offering prospects for the development of cross-coupling reactions through ancillary ligand design. The synthesis of PEPPSI-type catalysts is generally more straightforward, which is important for their use by a range of interested chemists. Furthermore, PEPPSI-type complexes are typically more cost-effective to prepare than other classes of Pd-NHC complexes, making them advantageous from a synthetic standpoint. Furthermore, activation to obtain active Pd(0)-NHC species follows different mechanisms for PEPPSI-type complexes, which provides different classes of sterically hindered complexes for cross-coupling reactions. Importantly, the present study demonstrates that the [IPr#–PEPPSI] complex can be employed for a range of N–C, O–C, C–Cl, C–Br, C–S and C–H bond activations.

To gain insight into the origin of the high catalytic reactivity of complex [IPr^#^–PEPPSI] (**1**), its electronic and steric properties were determined on the B3LYP 6-311++g(d,p) level.

**Figure 4:**
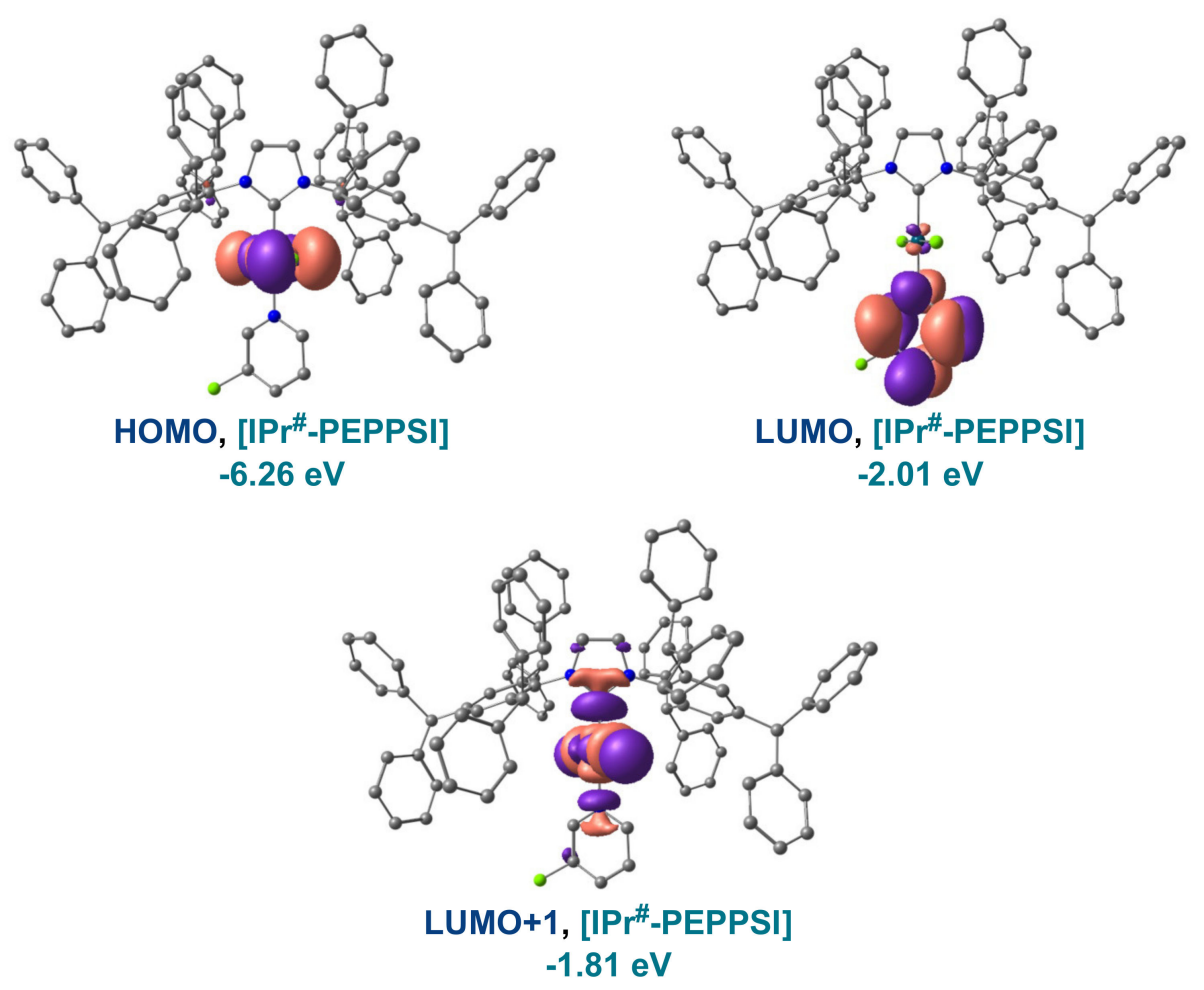
Frontier orbitals and energies (eV) of [IPr^#^–PEPPSI] (**1**) calculated on the level of B3LYP 6-311++g(d,p). See Supplementary Materials for details.

First, to gain insight into the electronic properties of [IPr^#^–PEPPSI] (**1**), the frontier molecular orbitals were calculated (Figure 4). The HOMO of [IPr^#^–PEPPSI] (**1**) (−6.26 eV) is located within Pd–Cl bonds. The energy HOMO of [IPr^#^–PEPPSI] (**1**) can be compared with the prototypical, less sterically demanding [IPr–PEPPSI] (−6.06 eV). Furthermore, the LUMO (−2.01 eV) and LUMO+1 (−1.81 eV) of the [IPr^#^–PEPPSI] (**1**) catalyst are lobed within Pd–N_py_ and Pd–C_NHC_ bonds, respectively. These values can be compared with the corresponding orbitals for [IPr–PEPPSI] (LUMO, −1.87 eV; LUMO+1, −1.63 eV). Overall, the HOMO and LUMO orbitals indicate a relative strength of the Pd–C_NHC_ bond compared with the Pd-N_py_ bond, with comparable levels of energy to IPr–PEPPSI, despite its much higher steric demand.

To gain further insight into the relative bond strengths, the Wiberg bond orders were determined (Table 2). The Wiberg bond orders of Pd–C_NHC_ (0.6801) and Pd–N_py_ (0.3099) for [IPr^#^–PEPPSI] (**1**) indicate a much stronger Pd–C_(carbene)_ bond vs. Pd–N_py_, which can be compared with the [IPr–PEPPSI] values of 0.6871 and 0.3267, respectively. Furthermore, the Pd–Cl1 (0.6062) and Pd–Cl2 (0.6032) bond orders of [IPr^#^–PEPPSI] (**1**) can be compared with the [IPr–PEPPSI] values of 0.6278 of 0.6302. Overall, the data indicate a strong Pd–C_(carbene)_ bond and capacity for the facile dissociation of ancillary and halide ligands in [IPr^#^–PEPPSI] (**1**).

**Figure 5:**
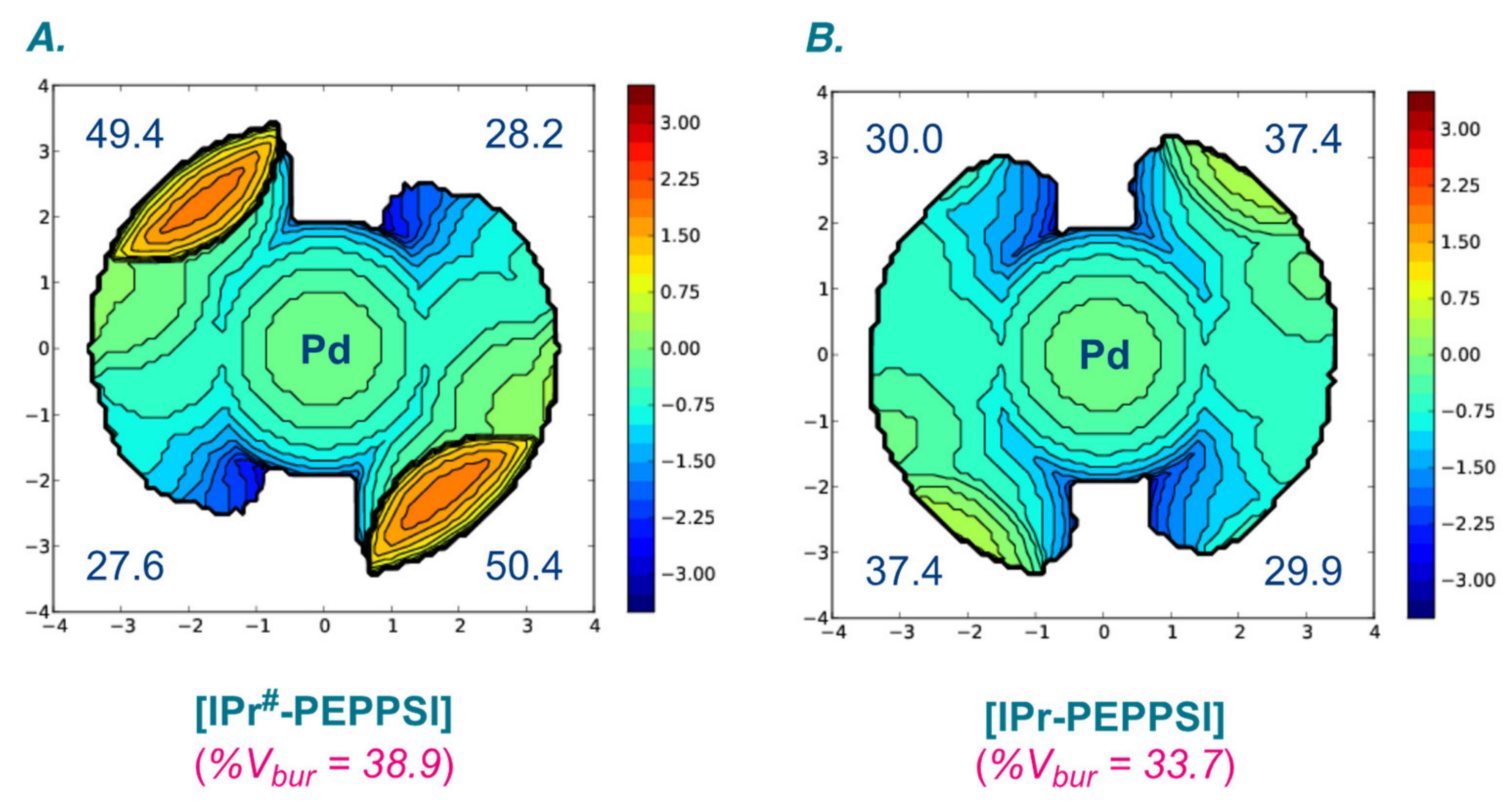
Topographical steric maps of [IPr^#^–PEPPSI] (**1**) and [IPr-PEPPSI] calculated on the B3LYP 6-311++g(d,p) level. (**A**) [IPr#-PEPPSI]; (**B**) [IPr-PEPPSI]. See Supplementary Materials for details.

Finally, to eliminate impacts of steric packing, the %V*_bur_* was calculated from the optimized structure of complex (**1**) on the B3LYP 6-311++g(d,p) level (Figure 5). The %V*_bur_* for [IPr^#^–PEPPSI] (**1**) is 38.9% (SW, 27.6%; NW, 49.4%; NE, 28.2%; SE, 50.4%), which can be compared with the [IPr–PEPPSI] %V*_bur_* of 33.7% (SW, 37.4%; NW, 30.0%; NE, 37.4%; SE, 29.9%). The data indicate a much greater steric impact of [IPr^#^–PEPPSI] (**1**) than of the classical IPr congener. Interestingly, [IPr^#^–PEPPSI] (**1**) features an asymmetrical distribution of the N-Ar wingtips, which is important in cross-coupling catalysis for the promotion of oxidative addition and reductive elimination steps. It should be noted that the steric map in Figure 3 is based on the crystal structure, and the steric map in Figure 5 is based on the computation. The steric distribution in Figure 3 is affected by crystal packing. The steric map in Figure 5 shows C2 symmetry in the %V*_bur_* calculation. The entire molecule is not C2-symmetric because we non-symmetric 3-Cl-py is present on the trans side.

Typically, a large steric %V*_bur_* of NHC ligands is required for effective cross-coupling reactions [25,26,27]. In general, ligands with %V*_bur_* of less than 40% are less efficient in cross-coupling due to their slower reductive elimination steps. Furthermore, it is interesting to note that ring-expanded NHCs show similar profiles to the present catalysts. For example, the six-membered analogue of IPr is characterized by a %V*_bur_* of 50.9%, and the seven-membered ring-expanded analogue of IPr is characterized by a %V*_bur_* of 52.7% [65].

## 3. Discussion

In conclusion, we reported on the synthesis, characterization and reactivity of [IPr^#^–PEPPSI]. This well-defined, air- and moisture-stable catalyst is based on a sterically demanding IPr^#^ framework obtained through the modular peralkylation of anilines. The broad activity of [IPr^#^–PEPPSI] in cross-coupling reactions with N–C, O–C, C–Cl, C–Br, C–S and C–H activations was demonstrated. A comprehensive evaluation of the steric and electronic properties provided further insight into the properties of [IPr^#^–PEPPSI]. Considering the easy accessibility of [IPr^#^–PEPPSI], its commercial availability (MilliporeSigma, 925489) and promising reactivity in a range of cross-coupling reactions, we expect that this class of catalysts will facilitate the broad use of Pd(II)–NHCs in catalysis research [63,64].

## 4. Materials and Methods

**General Procedure for the Preparation of [IPr^#^-PEPPSI] (1).** An oven-dried 10 mL vial equipped with a stir bar was charged with IPr^#^HCl (552 mg, 0.44 mmol, 1.1 equiv), PdCl_2_ (71 mg, 0.4 mmol, 1.0 equiv) and K_2_CO_3_ (276 mg, 2.0 mmol, 5.0 equiv). 3-Chloropyridine (2.0 mL) was added, and the reaction was stirred at 80 °C for 24 h. After cooling to room temperature, the mixture was diluted with DCM, and we filtered out the solid. The solution was collected and concentrated through evaporation in a high vacuum to remove the 3-Chloropyridine. The pure product was obtained via recrystallization in DCM/hexane as a white solid, with a yield of 82% (494 mg), as follows: ^1^H NMR (500 MHz, CDCl_3_) δ 8.97 (s, 1 H), 8.81 (d, *J* = 5.5 Hz, 1 H), 7.83 (d, *J* = 8.2 Hz, 1 H), 7.27 (m, 8 H), 7.15 (m, 12 H), 7.10–7.05 (m, 16 H), 7.00 (dd, *J* = 19.7, 7.4 Hz, 16 H), 6.78 (s, 4 H), 6.69 (d, *J* = 7.5 Hz, 8 H), 6.32 (s, 4 H), 5.38 (s, 2 H), 4.98 (s, 2 H); ^13^C NMR (125 MHz, CDCl_3_) δ 150.85, 149.93, 144.16, 144.04, 143.61, 141.79, 138.01, 135.88, 132.61, 131.42, 130.27, 129.47, 129.36, 128.27, 127.83, 126.23, 126.14, 126.06, 124.81, 124.14, 56.30, 51.09; HRMS (ESI) m/z: [M–Cl]+ Calcd for C_98_H_76_N_3_Cl_2_Pd 1472.4452, found 1472.4450. The crystallographic data were deposited in the Cambridge Crystallographic Data Center (CCDC 2262376).

**Activity of [Pd^#^-PEPPSI] in Cross-Coupling Reactions.** All cross-coupling reactions were carried out according to previously described procedures [13]. For comparative purposes, all products were identified via ^1^H NMR (500 MHz, CDCl_3_) and GC-MS using an internal standard and comparison with authentic samples. All yields correspond to yields determined via ^1^HNMR.

**N–C(O) Cleavage: Suzuki–Miyaura Amide Cross-Coupling.** An oven-dried vial equipped with a stir bar was charged with an amide substrate (29.7 mg, 0.10 mmol, 1.0 equiv), boronic acid (27.2 mg, 0.20 mmol, 2.0 equiv), potassium fluoride (17.4 mg, 0.30 mmol, 3.0 equiv) and [IPr^#^-PEPPSI] (3 mol%), placed under a positive pressure flow of argon, and subjected to three evacuation/backfilling cycles under a high vacuum. Toluene (0.40 mL, 0.25 M) and water (0.50 mmol, 5.0 equiv) were added with vigorous stirring at room temperature, and the reaction mixture was stirred at room temperature for 16 h. After the indicated time, the reaction mixture was diluted with CH_2_Cl_2_ (10 mL), filtered, and concentrated. The sample was analyzed via ^1^HNMR (CDCl_3_, 500 MHz) and GC-MS to obtain its conversion, selectivity and yield using an internal standard and comparison with authentic samples.

**O–C(O) Cleavage: Buchwald–Hartwig Ester Cross-Coupling.** An oven-dried vial equipped with a stir bar was charged with an ester substrate (19.8 mg, 0.10 mmol, 1.0 equiv), boronic acid (27.2 mg, 0.20 mmol, 2.0 equiv), potassium fluoride (17.4 mg, 0.30 mmol, 3.0 equiv), [IPr^#^-PEPPSI] (3 mol%), placed under a positive pressure flow of argon, and subjected to three evacuation/backfilling cycles under high vacuum. Toluene (0.40 mL, 0.25 M) and water (0.50 mmol, 5.0 equiv) were added with vigorous stirring at room temperature, and the reaction mixture was stirred at room temperature for 16 h. After the indicated time, the reaction mixture was diluted with CH_2_Cl_2_ (10 mL), filtered, and concentrated. The sample was analyzed via ^1^HNMR (CDCl_3_, 500 MHz) and GC-MS to obtain its conversion, selectivity and yield using an internal standard and comparison with authentic samples.

**N–C(O) Cleavage: Buchwald–Hartwig Amide Cross-Coupling (Transamidation).** An oven-dried vial equipped with a stir bar was charged with an amide substrate (29.7 mg, 0.10 mmol, 1.0 equiv), amine (24.6 mg, 0.20 mmol, 2.0 equiv), potassium carbonate (41.4 mg, 0.30 mmol, 3.0 equiv) and [IPr^#^-PEPPSI] (3 mol%), placed under a positive pressure of argon, and subjected to three evacuation/backfilling cycles under a high vacuum. DME (0.40 mL, 0.25 M) was added with vigorous stirring, and the reaction mixture was stirred at 110 °C for 16 h. After the indicated time, the reaction mixture was diluted with CH_2_Cl_2_ (10 mL), filtered, and concentrated. The sample was analyzed via ^1^HNMR (CDCl_3_, 500 MHz) and GC-MS to obtain the conversion, selectivity and yield using an internal standard and comparison with authentic samples.

**C–Cl Cleavage: Suzuki–Miyaura Cross-Coupling.** An oven-dried vial equipped with a stir bar was charged with an aryl chloride substrate (14.2 mg, 0.10 mmol, 1.0 equiv), boronic acid (27.2 mg, 0.20 mmol, 2.0 equiv), NaOH (12 mg, 0.30 mmol, 3.0 equiv) and [IPr^#^-PEPPSI] (1.0 mol%), placed under a positive pressure flow of argon, and subjected to three evacuation/backfilling cycles under a high vacuum. EtOH (0.40 mL, 0.25 M) was added with vigorous stirring at room temperature, and the reaction mixture was stirred at room temperature for 16 h. After the indicated time, the reaction mixture was diluted with CH_2_Cl_2_ (10 mL), filtered, and concentrated. The sample was analyzed via ^1^HNMR (CDCl_3_, 500 MHz) and GC-MS to obtain its conversion, selectivity and yield using an internal standard and comparison with authentic samples.

**C–Cl Cleavage: Buchwald–Hartwig Cross-Coupling.** An oven-dried vial equipped with a stir bar was charged with an aryl chloride substrate (14.2 mg, 0.10 mmol, 1.0 equiv), morpholine (17.4 mg, 0.20 mmol, 2.0 equiv) and [IPr^#^-PEPPSI] (3 mol%), placed under a positive pressure flow of argon, and subjected to three evacuation/backfilling cycles under a high vacuum. Dioxane (0.40 mL, 0.25 M) and LiHMDS (1.0 M in THF, 0.30 mmol, 3.0 equiv) were added with vigorous stirring at room temperature, and the reaction was stirred at 80 °C for 16 h. After the indicated time, the reaction mixture was diluted with CH_2_Cl_2_ (10 mL), washed with water (1 × 10 mL), extracted with CH_2_Cl_2_ (2 × 10 mL), dried over MgSO_4_, filtered and concentrated. The sample was analyzed via ^1^HNMR (CDCl_3_, 500 MHz) and GC-MS to obtain its conversion, selectivity and yield using an internal standard and comparison with authentic samples.

**C–Br Cleavage: Feringa Cross-Coupling with Aryllithium.** An oven-dried vial equipped with a stir bar was charged with an aryl bromide substrate (187 mg, 1.0 mmol, 1.0 equiv), PhLi (1.9 M in Bu_2_O, 2.0 mmol, 2.0 equiv) and [IPr^#^-PEPPSI] (2.5 mol%) at room temperature under argon and stirred for 10 min. After the indicated time, the reaction mixture was diluted with CH_2_Cl_2_ (10 mL), washed with water (1 × 10 mL), extracted with CH_2_Cl_2_ (2 × 10 mL), dried over MgSO_4_, filtered and concentrated. The sample was analyzed via ^1^HNMR (CDCl_3_, 500 MHz) and GC-MS to obtain its conversion, selectivity and yield using an internal standard and comparison with authentic samples.

**C–Br Cleavage: Feringa Cross-Coupling with Alkyllithium.** An oven-dried vial equipped with a stir bar was charged with an aryl bromide substrate (187 mg, 1.0 mmol, 1.0 equiv), *n*BuLi (2.5 M in hexanes, 2.0 mmol, 2.0 equiv) and [IPr^#^-PEPPSI] (2.5 mol%) at room temperature under argon and stirred for 10 min. After the indicated time, the reaction mixture was diluted with CH_2_Cl_2_ (10 mL), washed with water (1 × 10 mL), extracted with CH_2_Cl_2_ (2 × 10 mL), dried over MgSO_4_, filtered and concentrated. The sample was analyzed via ^1^HNMR (CDCl_3_, 500 MHz) and GC-MS to obtain its conversion, selectivity and yield using an internal standard and comparison with authentic samples.

**C–Cl Cleavage: α-Ketone Arylation.** An oven-dried vial equipped with a stir bar was charged with a ketone substrate (13.4 mg, 0.10 mmol, 1.0 equiv), chlorobenzene (22.4 mg, 0.20 mmol, 2.0 equiv) and [IPr^#^-PEPPSI] (3 mol%), placed under a positive pressure flow of argon, and subjected to three evacuation/backfilling cycles under high vacuum. Toluene (0.40 mL, 0.25 M) and LiHMDS (1.0 M in THF, 0.20 mmol, 2.0 equiv) were added with vigorous stirring at room temperature, and the reaction mixture was stirred at 100 °C for 24 h. After the indicated time, the reaction mixture was diluted with CH_2_Cl_2_ (10 mL), washed with water (1 × 10 mL), extracted with CH_2_Cl_2_ (2 × 10 mL), dried over MgSO_4_, filtered and concentrated. The sample was analyzed via ^1^HNMR (CDCl_3_, 500 MHz) and GC-MS to obtain its conversion, selectivity and yield using an internal standard and comparison with authentic samples.

**C–S Cleavage: Carbon–Sulfur Bond Metathesis.** An oven-dried vial equipped with a stir bar was charged with a thioether substrate (12.4 mg, 0.10 mmol, 1.0 equiv), cyclohexanethiol (26.0 mg, 0.20 mmol, 2.0 equiv) and [IPr^#^-PEPPSI] (3 mol%), placed under a positive pressure flow of argon, and subjected to three evacuation/backfilling cycles under a high vacuum. Toluene (0.10 mL, 1.0 M) and LiHMDS (1.0 M in THF, 0.26 mmol, 2.6 equiv) were added with vigorous stirring at room temperature, and the reaction mixture was stirred at 110 °C for 16 h. After the indicated time, the reaction mixture was diluted with CH_2_Cl_2_ (10 mL), washed with water (1 × 10 mL), extracted with CH_2_Cl_2_ (2 × 10 mL), dried over MgSO_4_, filtered and concentrated. The sample was analyzed via ^1^HNMR (CDCl_3_, 500 MHz) and GC-MS to obtain its conversion, selectivity and yield using an internal standard and comparison with authentic samples.

**C–H Cleavage: Direct C–H Arylation.** An oven-dried vial equipped with a stir bar was charged with a thiophene substrate (9.8 mg, 0.10 mmol, 1.0 equiv), 1-bromo-4-methylbenzene (18.8 mg, 0.11 mmol, 1.1 equiv), potassium carbonate (20.7 mg, 0.15 mmol, 1.5 equiv), PivOH (3.1 mg, 0.03 mmol, 0.30 equiv) and [IPr^#^-PEPPSI] (0.1 mol%), placed under a positive pressure flow of argon, and subjected to three evacuation/backfilling cycles under a high vacuum. DMA (0.40 mL, 0.25 M) was added with vigorous stirring at room temperature, and the reaction mixture was stirred at 140 °C for 16 h. After the indicated time, the reaction mixture was diluted with CH_2_Cl_2_ (10 mL), washed with water (1 × 10 mL), extracted with CH_2_Cl_2_ (2 × 10 mL), dried over MgSO_4_, filtered and concentrated. The sample was analyzed via ^1^HNMR (CDCl_3_, 500 MHz) and GC-MS to obtain its conversion, selectivity and yield using an internal standard and comparison with authentic samples.

**Crystallographic Analysis.** The crystal data and structure refinement summaries for [IPr#-PEPPSI] are included in Table S1 in the Supplementary Materials. The Supplementary Materials also include the large ORTEP structures of [IPr#-PEPPSI] (50% ellipsoids) in the NHC–M parallel plane and NHC–M perpendicular plane.

**Computational Methods.** All the calculations were performed using the Gaussian 09 suite of programs. All of the geometry optimizations were performed on the B3LYP level of theory in the gas phase with the QZVP basis set for palladium and the 6-311++G(d,p) basis set for the other atoms. For the geometry optimizations, we employed the X-ray structure of [IPr^#^–PEPPSI] as the starting geometry and performed full optimization. The absence of imaginary frequencies was used to characterize the structures as minima on the potential energy surface. All of the optimized geometries were verified as minima (no imaginary frequencies). NBO calculations were performed on the DFT/B3LYP level using the NBO program implemented in the Gaussian software package. The Wiberg bond indices were calculated using the NBO method. The energetic parameters were calculated under standard conditions (298.15 K and 1 atm). The structural representations were generated using CYLview software (Legault, C. Y. CYL view version 1.0 BETA, University of Sherbrooke). All other representations were generated using Gauss View (GaussView, version 5, Dennington, R.; Keith, T.; Millam, J. Semichem Inc., Shawnee Mission, KS, 2009) or ChemCraft software (Andrienko, G. L. ChemCraft version b562a).

## Figures and Tables

**Table 1:** Comparison of the percent buried volume (%V*_bur_*) and bond lengths of [Pd-NHC] complexes ^1^.

Entry	Complexes	%V*_bur_*	Pd–C_NHC_ (Å)	Pd–N_py_ (Å)	Pd–Cl (Å)
1	**[IPr^#^–PEPPSI]** (*molecule-1*)	40.2	1.978(4)	2.119(4)	2.285(1), 2.300(1)
2	**[IPr^#^–PEPPSI]** (*molecule-2*)	38.2	1.965(4)	2.114(4)	2.301(1), 2.302(1)
3	**[IPr–PEPPSI]**	34.8	1.969(3)	2.137(3)	2.290(9), 2.298(7)
4	**[IPent–PEPPSI]**	38.3	1.975(3)	2.097(2)	2.2868(9), 2.3033(9)
5	**[Pd(IPr^#^)(cin)Cl]**	44.7	2.046(4)	2.117(5), 2.133(6), 2.216(7) *^a^*	2.374(1)

^1^ X-ray single-crystal structure data. *^a^* Pd–cin bond lengths (Å).

**Table 2:** Bond orders of [IPr^#^-PEPPSI] (**1**) and [IPr–PEPPSI] complexes ^1^.

Entry	Complexes	Pd–C_NHC_	Pd–N_py_	Pd–Cl
1	**[IPr^#^–PEPPSI]**	0.6801	0.3099	0.6062, 0.6032
2	**[IPr–PEPPSI]**	0.6871	0.3267	0.6278, 0.6302

^1^ Calculated on the B3LYP 6-311++g(d,p) level.

## Data Availability

Data is contained within the article or Supplementary Materials.

## Supplementary Materials

Procedures and computational data are available online at https://www.mdpi.com/article/10.3390/molecules28155833/s1. Table S1: Crystal Data and Structure Refinement Summaries for [IPr#-PEPPSI] (1). Figures S1–S4: ORTEP Structure of [IPr#-PEPPSI] (1) (50% ellipsoids). Figure S5: Topographical Steric Maps of [IPr#-PEPPSI] (1); Figure S6: Graphical Representation of Frontier Orbitals of Complexes [IPr#-PEPPSI] and [IPr-PEPPSI] at the B3LYP 6-311++g(d,p) Level; Figure S7: Topographical Steric Maps of Complexes [IPr#-PEPPSI] and [IPr-PEPPSI] at the B3LYP 6-311++g(d,p) Level. Table S2: Steric Parameters of Pd–NHC Complexes Calculated from X-ray Crystallography; Table S3: Frontier Orbitals and Energy of Complexes [IPr#-PEPPSI] and [IPr-PEPPSI] Calculated at the B3LYP 6-311++g(d,p) Level; Table S4: Steric Parameters of Complexes [IPr#-PEPPSI] and [Ipr-PEPPSI] Calculated at the B3LYP 6-311++g(d,p) Level [66].
