# Supplementary material for: [IPr#–PEPPSI]: A Well-Defined, Highly Hindered and Broadly Applicable Pd(II)–NHC (NHC = N-Heterocyclic Carbene) Precatalyst for Cross-Coupling Reactions
Source: Molecules. 2023 Aug 2;28(15):5833. doi: 10.3390/molecules28155833 (PMC10421006; doi:10.3390/molecules28155833)
Supplement: Supplementary file 1 [file molecules-28-05833-s001.zip › molecules-2503482-supplementary.pdf]

Supporting Information

[IPr#–PEPPSI] – Well-Defined, Highly Hindered and Broadly Applicable Pd(II)–NHC (NHC = N-Heterocyclic Carbene) Precatalyst for Cross-Coupling Reactions

Md. Mahbubur Rahman,[†,§] Qun Zhao,[†,§] Guangrong Meng[†,§] Roger Lalancette,[†] Roman Szostak[‡] and Michal Szostak*,[†]

[†]*Department of Chemistry, Rutgers University, 73 Warren St, Newark, New Jersey 07102, USA*
[‡]*Department of Chemistry, Wroclaw University, F. Joliot-Curie 14, Wroclaw 50-383, Poland*

michal.szostak@rutgers.edu

Table of Contents S1

List of Known Compounds/General Methods S2
Experimental Procedures and Characterization Data S3
Crystallographic Studies S9
Computational Methods S16
^{1}H and ^{13}C NMR Spectra S22
Cartesian Coordinates with Zero-Point Energies and Thermal Corrections S23

Corresponding Author:
Prof. Dr. M. Szostak
Department of Chemistry, Rutgers University
73 Warren Street, Newark, NJ 07102, United States
E-mail: michal.szostak@rutgers.edu
[§]These authors contributed equally

List of Known Compounds/General Methods

All solvents were purchased at the highest commercial grade and used as received or after purification by passing through activated alumina columns or distillation from sodium/benzophenone under nitrogen. All solvents were deoxygenated prior to use. All other chemicals were purchased at the highest commercial grade and used as received. Reaction glassware was oven-dried at 140 °C for at least 24 h or flame-dried prior to use, allowed to cool under vacuum and purged with argon (three cycles). All products were identified using ^{1}H NMR analysis and comparison with authentic samples. ^{1}H NMR and ^{13}C NMR spectra were recorded in $CDCl_3$ on Bruker spectrometers at 500 (^{1}H NMR) and 125 MHz (^{13}C NMR). All shifts are reported in parts per million (ppm) relative to residual $CHCl_3$ peak (7.27 and 77.2 ppm, ^{1}H NMR and ^{13}C NMR, respectively). All coupling constants (J) are reported in hertz (Hz). Abbreviations are: s, singlet; d, doublet; t, triplet; q, quartet; brs, broad singlet. High-resolution mass spectra (HRMS) were measured on a 7T Bruker Daltonics FT-MS instrument (for HRMS). Melting point was measured on MeltEMP (laboratory devices). GC-MS chromatography was performed using Agilent HP6890 GC System and Agilent 5973A inert XL EI/CI MSD using helium as the carrier gas at a flow rate of 1 mL/min and an initial oven temperature of 50 °C. The injector temperature was 250 °C. The detector temperature was 250 °C. For runs with the initial oven temperature of 50 °C, temperature was increased with a 10 °C/min ramp after 50 °C hold for 3 min to a final temperature of 220 °C, then hold at 220 °C for 15 min (splitless mode of injection, total run time of 22.0 min). All flash chromatography was performed using silica gel, 60 Å, 300 mesh. TLC analysis was carried out on aluminium sheets coated with silica gel 60 F254, 0.2 mm thickness. The plates were visualized using a 254 nm ultraviolet lamp or aqueous potassium permanganate solutions.

Experimental Procedures and Characterization Data

A. Synthesis of [IPr#-PEPPSI] (1).

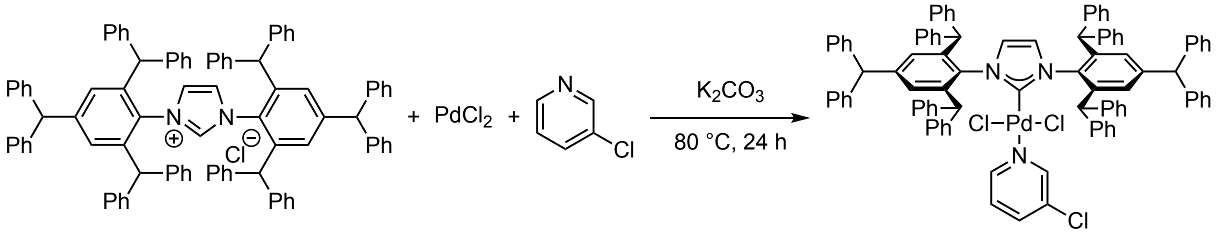

An oven-dried 10 mL vial equipped with a stir bar was charged with IPr#HCl (**1**) (552 mg, 0.44 mmol, 1.1 equiv), $PdCl_2$ (71 mg, 0.4 mmol, 1.0 equiv), K_2CO_3 (276 mg, 2.0 mmol, 5.0 equiv). 3-Chloropyridine (2.0 ml) was added, and the reaction was stirred at 80 °C for 24 h. After cooling to room temperature, the mixture was diluted with DCM and filtered out the solid. The solution was collected and concentrated by evaporation and high vacuum to remove the 3-Chloropyridine. The pure product was obtained by recrystallization in DCM/hexane as a white solid. Yield 82% (494 mg). ^{1}H NMR (500 MHz, $CDCl_3$) δ 8.97 (s, 1 H), 8.81 (d, *J* = 5.5 Hz, 1 H), 7.83 (d, *J* = 8.2 Hz, 1 H), 7.27 (m, 8 H), 7.15 (m, 12 H), 7.10-7.05 (m, 16 H), 7.00 (dd, *J* = 19.7, 7.4 Hz, 16 H), 6.78 (s, 4 H), 6.69 (d, *J* = 7.5 Hz, 8 H), 6.32 (s, 4 H), 5.38 (s, 2 H), 4.98 (s, 2 H). ^{13}C NMR (125 MHz, $CDCl_3$) δ 150.85, 149.93, 144.16, 144.04, 143.61, 141.79, 138.01, 135.88, 132.61, 131.42, 130.27, 129.47, 129.36, 128.27, 127.83, 126.23, 126.14, 126.06, 124.81, 124.14, 56.30, 51.09. HRMS (ESI) m/z: $[M – Cl]^+$ Calcd for $C_{98}H_{76}N_3Cl_2Pd$ 1472.4452, found 1472.4450.

B. Activity of [Pd#-PEPPSI] in Cross-Coupling Reactions

All cross-coupling reactions were carried out according to the procedures previously described (Zhao, Q.; Meng, G.; Li, G.; Flach, C.; Mendelsohn, R.; Lalancette, R.; Szostak, R.; Szostak, M. *Chem. Sci.* **2021**, *12*, 10583-10589). For comparison purposes, all products were identified by ^{1}H NMR (500 MHz, $CDCl_3$) and GC-MS using internal standard and comparison with authentic samples. All yields correspond to yields determined by ^{1}HNMR.

1. N–C(O) Cleavage: Suzuki–Miyaura Amide Cross-Coupling

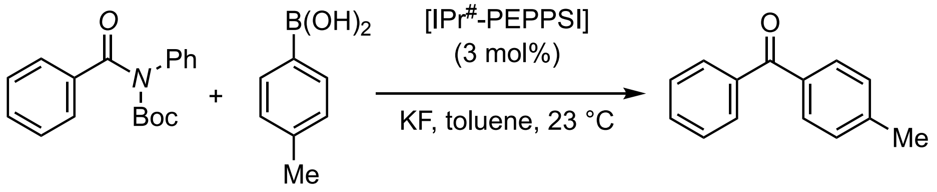

An oven dried vial equipped with a stir bar was charged with an amide substrate (29.7 mg, 0.10 mmol, 1.0 equiv), boronic acid (27.2 mg, 0.20 mmol, 2.0 equiv), potassium fluoride (17.4 mg, 0.30 mmol, 3.0 equiv), [IPr#-PEPPSI] (3 mol%), placed under a positive pressure of argon, and subjected to three evacuation/backfilling cycles under high vacuum. Toluene (0.40 mL, 0.25 M) and water (0.50 mmol, 5.0 equiv) were added with vigorous stirring at room temperature, and the reaction mixture was stirred at room temperature for 16 h. After the indicated time, the reaction mixture was diluted with CH_2Cl_2 (10 mL), filtered, and concentrated. The sample was analyzed by ^{1}HNMR ($CDCl_3$, 500 MHz) and GC-MS to obtain conversion, selectivity and yield using internal standard and comparison with authentic samples.

2. O–C(O) Cleavage: Buchwald-Hartwig Ester Cross-Coupling

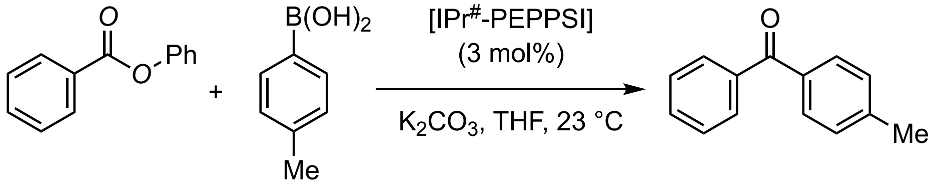

An oven dried vial equipped with a stir bar was charged with an ester substrate (19.8 mg, 0.10 mmol, 1.0 equiv), boronic acid (27.2 mg, 0.20 mmol, 2.0 equiv), potassium fluoride (17.4 mg, 0.30 mmol, 3.0 equiv), [IPr#-PEPPSI] (3 mol%), placed under a positive pressure of argon, and subjected to three evacuation/backfilling cycles under high vacuum. Toluene (0.40 mL, 0.25 M)

and water (0.50 mmol, 5.0 equiv) were added with vigorous stirring at room temperature, and the reaction mixture was stirred at room temperature for 16 h. After the indicated time, the reaction mixture was diluted with CH_2Cl_2 (10 mL), filtered, and concentrated. The sample was analyzed by ^{1}HNMR ($CDCl_3$, 500 MHz) and GC-MS to obtain conversion, selectivity and yield using internal standard and comparison with authentic samples.

3. N–C(O) Cleavage: Buchwald-Hartwig Amide Cross-Coupling (Transamidation)

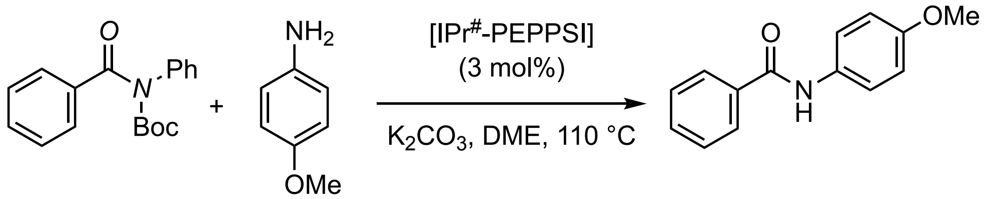

An oven dried vial equipped with a stir bar was charged with an amide substrate (29.7 mg, 0.10 mmol, 1.0 equiv), amine (24.6 mg, 0.20 mmol, 2.0 equiv), potassium carbonate (41.4 mg, 0.30 mmol, 3.0 equiv), [IPr$^\#$-PEPPSI] (3 mol%), placed under a positive pressure of argon, and subjected to three evacuation/backfilling cycles under high vacuum. DME (0.40 mL, 0.25 M) was added with vigorous stirring and the reaction mixture was stirred at 110 °C for 16 h. After the indicated time, the reaction mixture was diluted with CH_2Cl_2 (10 mL), filtered, and concentrated. The sample was analyzed by ^{1}HNMR ($CDCl_3$, 500 MHz) and GC-MS to obtain conversion, selectivity and yield using internal standard and comparison with authentic samples.

4. C–Cl Cleavage: Suzuki–Miyaura Cross-Coupling

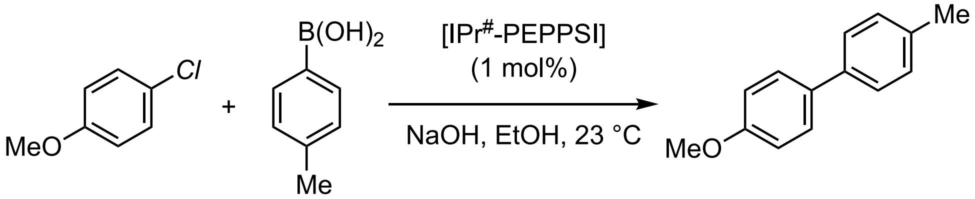

An oven dried vial equipped with a stir bar was charged with an aryl chloride substrate (14.2 mg, 0.10 mmol, 1.0 equiv), boronic acid (27.2 mg, 0.20 mmol, 2.0 equiv), NaOH (12 mg, 0.30 mmol, 3.0 equiv), [IPr$^\#$-PEPPSI] (1.0 mol%), placed under a positive pressure of argon, and subjected to three evacuation/backfilling cycles under high vacuum. EtOH (0.40 mL, 0.25 M) was added with vigorous stirring at room temperature and the reaction mixture was stirred at room temperature for 16 h. After the indicated time, the reaction mixture was diluted with CH_2Cl_2 (10 mL), filtered, and

concentrated. The sample was analyzed by ^{1}HNMR ($CDCl_3$, 500 MHz) and GC-MS to obtain conversion, selectivity and yield using internal standard and comparison with authentic samples.

5. C–Cl Cleavage: Buchwald-Hartwig Cross-Coupling

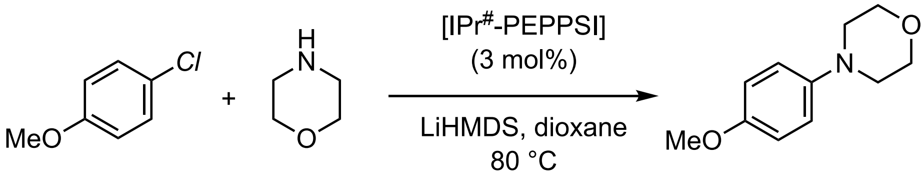

An oven dried vial equipped with a stir bar was charged with an aryl chloride substrate (14.2 mg, 0.10 mmol, 1.0 equiv), morpholine (17.4 mg, 0.20 mmol, 2.0 equiv), [IPr#-PEPPSI] (3 mol%), placed under a positive pressure of argon, and subjected to three evacuation/backfilling cycles under high vacuum. Dioxane (0.40 mL, 0.25 M) was added and LiHMDS (1.0 M in THF, 0.30 mmol, 3.0 equiv) were added with vigorous stirring at room temperature and the reaction was stirred at 80 °C for 16 h. After the indicated time, the reaction mixture was diluted with CH_2Cl_2 (10 mL), washed with water (1 x 10 mL), extracted with CH_2Cl_2 (2 x 10 mL), dried over $MgSO_4$, filtered and concentrated. The sample was analyzed by ^{1}HNMR ($CDCl_3$, 500 MHz) and GC-MS to obtain conversion, selectivity and yield using internal standard and comparison with authentic samples.

6. C–Br Cleavage: Feringa Cross-Coupling with Aryllithium

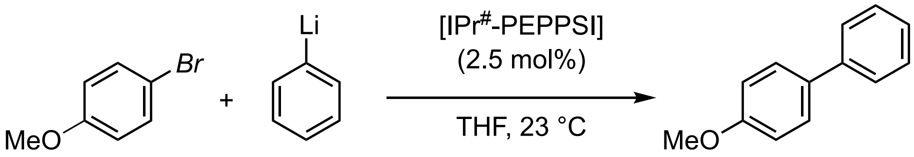

An oven dried vial equipped with a stir bar was charged with an aryl bromide substrate (187 mg, 1.0 mmol, 1.0 equiv), PhLi (1.9 M in Bu_2O, 2.0 mmol, 2.0 equiv) and [IPr#-PEPPSI] (2.5 mol%) at room temperature under argon and stirred for 10 min. After the indicated time, the reaction mixture was diluted with CH_2Cl_2 (10 mL), washed with water (1 x 10 mL), extracted with CH_2Cl_2 (2 x 10 mL), dried over $MgSO_4$, filtered and concentrated. The sample was analyzed by ^{1}HNMR ($CDCl_3$, 500 MHz) and GC-MS to obtain conversion, selectivity and yield using internal standard and comparison with authentic samples.

7. C–Br Cleavage: Feringa Cross-Coupling with Alkyllithium

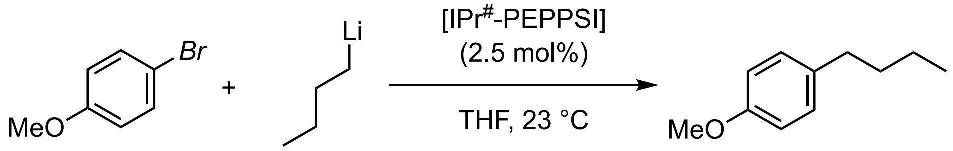

An oven dried vial equipped with a stir bar was charged with an aryl bromide substrate (187 mg, 1.0 mmol, 1.0 equiv), *n*BuLi (2.5 M in hexanes, 2.0 mmol, 2.0 equiv) and [IPr#-PEPPSI] (2.5 mol%) at room temperature under argon and stirred for 10 min. After the indicated time, the reaction mixture was diluted with CH_2Cl_2 (10 mL), washed with water (1 x 10 mL), extracted with CH_2Cl_2 (2 x 10 mL), dried over $MgSO_4$, filtered and concentrated. The sample was analyzed by ^{1}HNMR ($CDCl_3$, 500 MHz) and GC-MS to obtain conversion, selectivity and yield using internal standard and comparison with authentic samples.

8. C–Cl Cleavage: α-Ketone Arylation

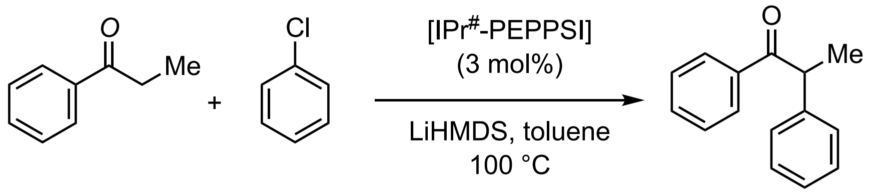

An oven dried vial equipped with a stir bar was charged with a ketone substrate (13.4 mg, 0.10 mmol, 1.0 equiv), chlorobenzene (22.4 mg, 0.20 mmol, 2.0 equiv), [IPr#-PEPPSI] (3 mol%), placed under a positive pressure of argon, and subjected to three evacuation/backfilling cycles under high vacuum. Toluene (0.40 mL, 0.25 M) and LiHMDS (1.0 M in THF, 0.20 mmol, 2.0 equiv) were added with vigorous stirring at room temperature and the reaction mixture was stirred at 100 °C for 24 h. After the indicated time, the reaction mixture was diluted with CH_2Cl_2 (10 mL), washed with water (1 x 10 mL), extracted with CH_2Cl_2 (2 x 10 mL), dried over $MgSO_4$, filtered and concentrated. The sample was analyzed by ^{1}HNMR ($CDCl_3$, 500 MHz) and GC-MS to obtain conversion, selectivity and yield using internal standard and comparison with authentic samples.

9. C–S Cleavage: Carbon–Sulfur Bond Metathesis

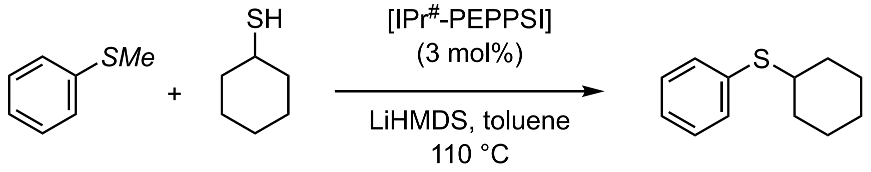

An oven dried vial equipped with a stir bar was charged with a thioether substrate (12.4 mg, 0.10 mmol, 1.0 equiv), cyclohexanethiol (26.0 mg, 0.20 mmol, 2.0 equiv), [IPr#-PEPPSI] (3 mol%), placed under a positive pressure of argon, and subjected to three evacuation/backfilling cycles under high vacuum. Toluene (0.10 mL, 1.0 M) and LiHMDS (1.0 M in THF, 0.26 mmol, 2.6 equiv) were added with vigorous stirring at room temperature and the reaction mixture was stirred at 110 °C for 16 h. After the indicated time, the reaction mixture was diluted with CH_2Cl_2 (10 mL), washed with water (1 x 10 mL), extracted with CH_2Cl_2 (2 x 10 mL), dried over $MgSO_4$, filtered and concentrated. The sample was analyzed by ^{1}HNMR ($CDCl_3$, 500 MHz) and GC-MS to obtain conversion, selectivity and yield using internal standard and comparison with authentic samples.

10. C–H Cleavage: Direct C–H Arylation

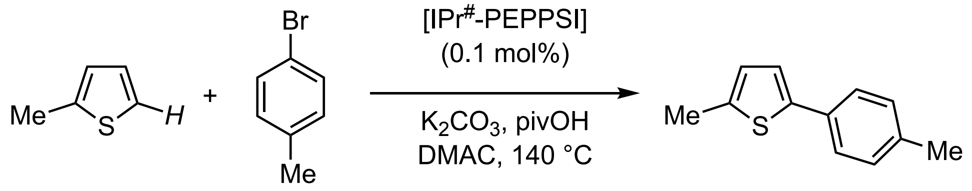

An oven dried vial equipped with a stir bar was charged with a thiophene substrate (9.8 mg, 0.10 mmol, 1.0 equiv), 1-bromo-4-methylbenzene (18.8 mg, 0.11 mmol, 1.1 equiv), potassium carbonate (20.7 mg, 0.15 mmol, 1.5 equiv), PivOH (3.1 mg, 0.03 mmol, 0.30 equiv), [IPr#-PEPPSI] (0.1 mol%), placed under a positive pressure of argon, and subjected to three evacuation/backfilling cycles under high vacuum. DMA (0.40 mL, 0.25 M) was added with vigorous stirring at room temperature and the reaction mixture was stirred at 140 °C for 16 h. After the indicated time, the reaction mixture was diluted with CH_2Cl_2 (10 mL), washed with water (1 x 10 mL), extracted with CH_2Cl_2 (2 x 10 mL), dried over $MgSO_4$, filtered and concentrated. The sample was analyzed by ^{1}HNMR ($CDCl_3$, 500 MHz) and GC-MS to obtain conversion, selectivity and yield using internal standard and comparison with authentic samples.

C. Crystallographic Analysis

Table S1. *Crystal Data and Structure Refinement Summaries for* ***[IPr#-PEPPSI] (1)***.

**Compound**	**[IPr#-PEPPSI] (1)**
Chemical formula	$2(C_{98}H_{76}Cl_3N_3Pd)\cdot 3(CH_2Cl_2)$
M_r	3271.52
Crystal system, space group	Triclinic, *P*1
Temperature (K)	100
a, *b*, *c* (Å)	11.5433 (2), 25.8068 (4), 28.3687 (4)
α, β, γ (°)	107.499 (1), 90.691 (1), 93.344 (1)
V (Å^3)	8042.2 (2)
Z	2
Radiation type	Cu *K*α
μ (mm^{-1})	4.09
Crystal size (mm)	0.27 × 0.19 × 0.10
Data collection	
Diffractometer	Bruker *SMART* CCD Apex-II area-detector
Absorption correction	Numerical<br>*SADABS* (Sheldrick, 2008a)
T_{min}, T_{max}	0.530, 0.789
No. of measured, independent and observed [$I > 2\sigma(I)$] reflections	72943, 26832, 20529
R_{int}	0.039
$(\sin \theta/\lambda)_{max}$ (Å^{-1})	0.602
Refinement	
$R[F^2 > 2\sigma(F^2)]$, $wR(F^2)$, S	0.058, 0.163, 1.02
No. of reflections	26832
No. of parameters	1972
H-atom treatment	H-atom parameters constrained
	$w = 1/[\sigma^2(F_o^2) + (0.0743P)^2 + 22.868P]$<br>where $P = (F_o^2 + 2F_c^2)/3$
$\Delta\rho_{max}$, $\Delta\rho_{min}$ (e Å^{-3})	1.54, −1.31

Computer programs: *APEX* 2 (Bruker, 2006), *APEX* 2, *SAINT* (Bruker, 2005), *SHELXL2014*/7 (Sheldrick, 2014), *SHELXTL*.

Figure S1. *ORTEP Structure of* ***[IPr#-PEPPSI] (1)*** *(50% ellipsoids). (Crystallographic data has been deposited with the Cambridge Crystallographic Data Center as supplementary publication no. CCDC 2262376).*

molecule-1: NHC–M parallel plane

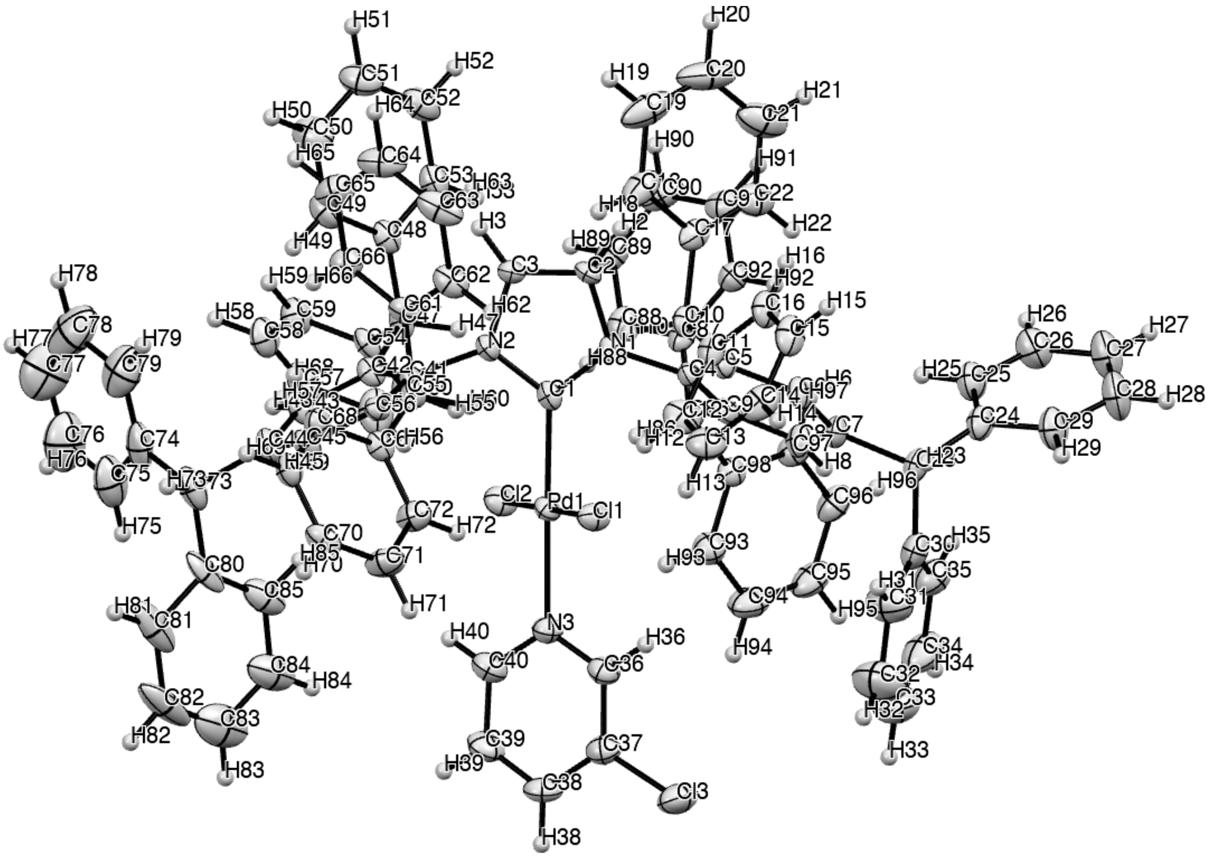

Selected bond lengths [Å], bond angle [°] and dihedral angles [°] (**2**): Pd1–C1, 1.978(4); Pd1–Cl1, 2.285(1); Pd1–Cl2, 2.300(1); Pd1–N3, 2.119(4); C1–N1, 1.353(6); C1–N2, 1.356(6); C1–Pd1–Cl1, 88.8(1); C1–Pd1–Cl2, 91.5(1); N3–Pd1–Cl1, 90.6(1); N3–Pd1–Cl2, 89.1(1).

Figure S2. *ORTEP Structure of* ***[IPr#-PEPPSI] (1)*** *(50% ellipsoids). (Crystallographic data has been deposited with the Cambridge Crystallographic Data Center as supplementary publication no. CCDC 2262376).*

molecule-1: NHC–M perpendicular plane

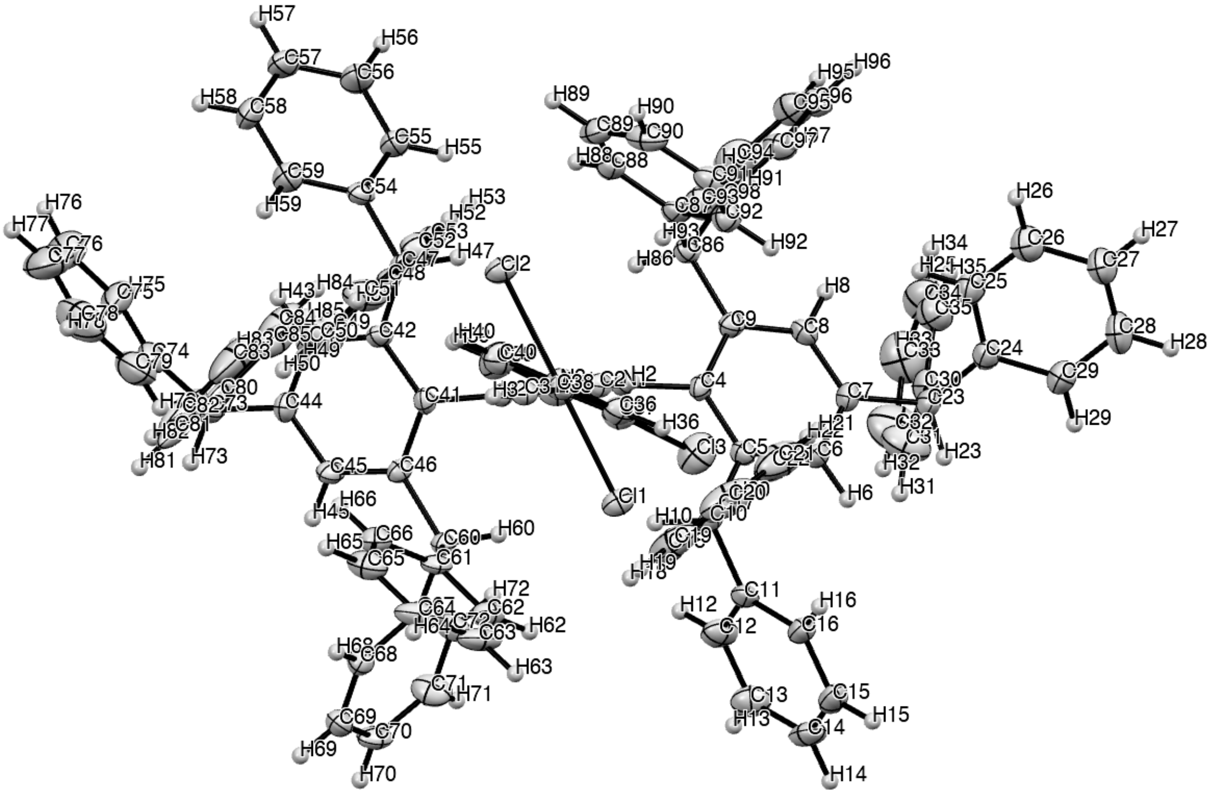

Selected bond lengths [Å], bond angle [°] and dihedral angles [°] (**2**): Pd1–C1, 1.978(4); Pd1–Cl1, 2.285(1); Pd1–Cl2, 2.300(1); Pd1–N3, 2.119(4); C1–N1, 1.353(6); C1–N2, 1.356(6); C1–Pd1–Cl1, 88.8(1); C1–Pd1–Cl2, 91.5(1); N3–Pd1–Cl1, 90.6(1); N3–Pd1–Cl2, 89.1(1).

Figure S3. *ORTEP Structure of* ***[IPr#-PEPPSI] (1)*** *(50% ellipsoids). (Crystallographic data has been deposited with the Cambridge Crystallographic Data Center as supplementary publication no. CCDC 2262376).*

molecule-2: NHC–M parallel plane

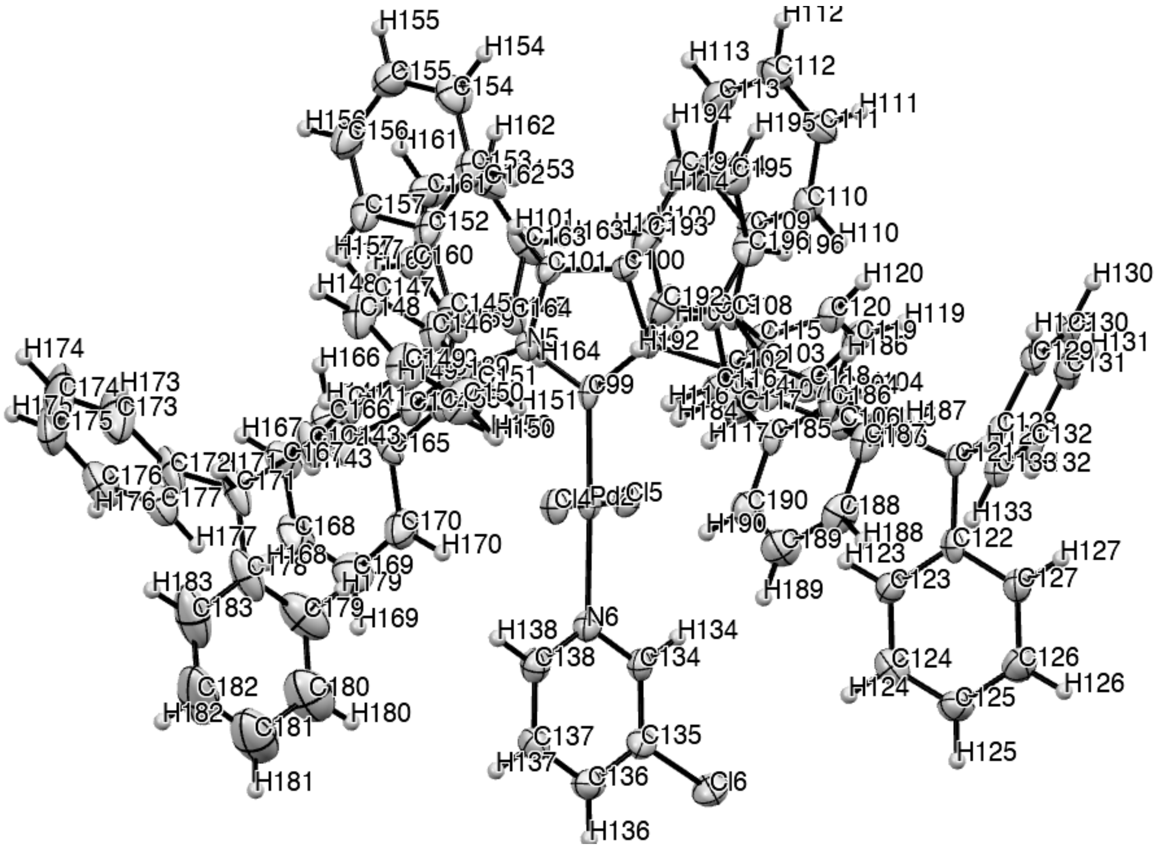

Selected bond lengths [Å], bond angle [°] and dihedral angles [°] (**2**): Pd2–C99, 1.965(4); Pd2–Cl14, 2.301(1); Pd2–Cl15, 2.302(1); Pd2–N6, 2.114(4); C99–N4, 1.365(6); C99–N5, 1.354(6); C99–Pd2–Cl14, 89.8(1); C99–Pd2–Cl15, 89.4(1); N6–Pd2–Cl14, 90.4(1); N6–Pd2–Cl15, 90.4(1).

Figure S4. *ORTEP Structure of* ***[IPr#-PEPPSI] (1)*** *(50% ellipsoids). (Crystallographic data has been deposited with the Cambridge Crystallographic Data Center as supplementary publication no. CCDC 2262376).*

molecule-2: NHC–M perpendicular plane

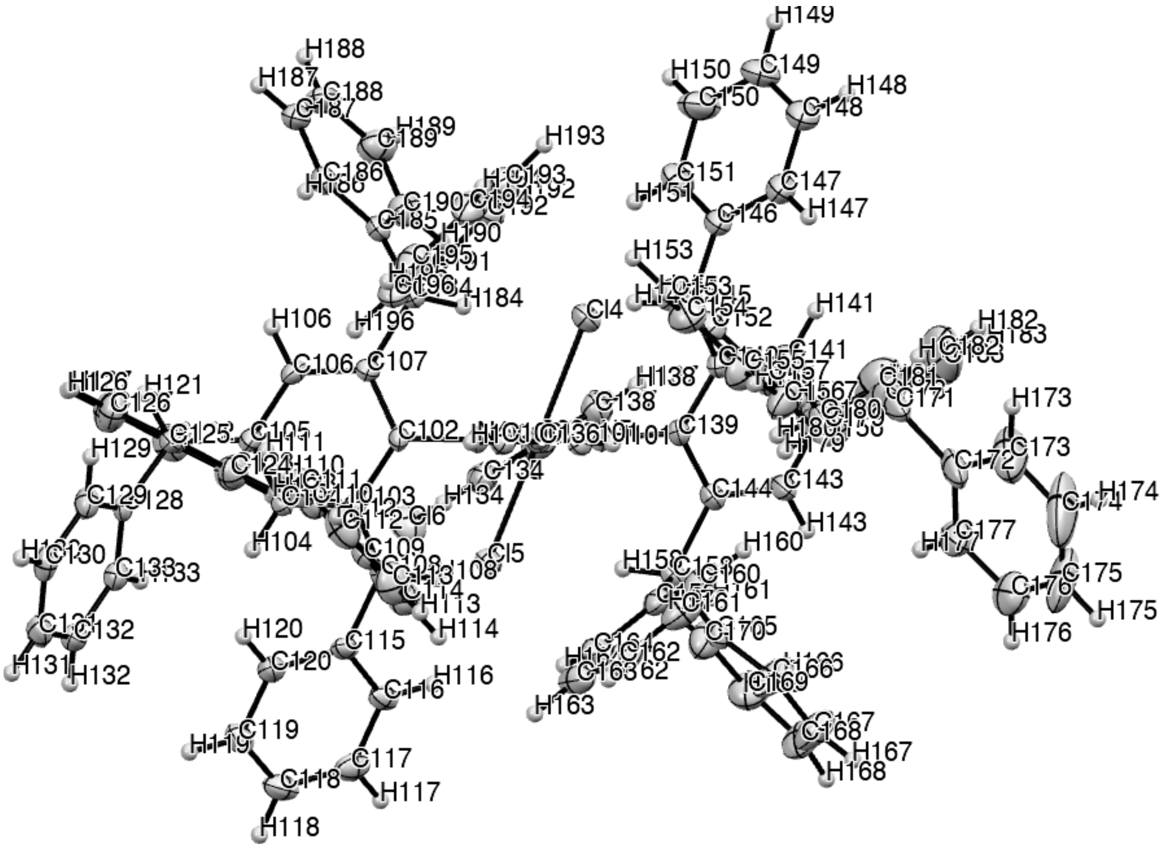

Selected bond lengths [Å], bond angle [°] and dihedral angles [°] (**2**): Pd2–C99, 1.965(4); Pd2–Cl14, 2.301(1); Pd2–Cl15, 2.302(1); Pd2–N6, 2.114(4); C99–N4, 1.365(6); C99–N5, 1.354(6); C99–Pd2–Cl14, 89.8(1); C99–Pd2–Cl15, 89.4(1); N6–Pd2–Cl14, 90.4(1); N6–Pd2–Cl15, 90.4(1).

Figure S5. *Topographical Steric Maps of* ***[IPr#-PEPPSI] (1)*** *Calculated from X-ray Crystal Structure Showing %V_{bur} per Quadrant.*

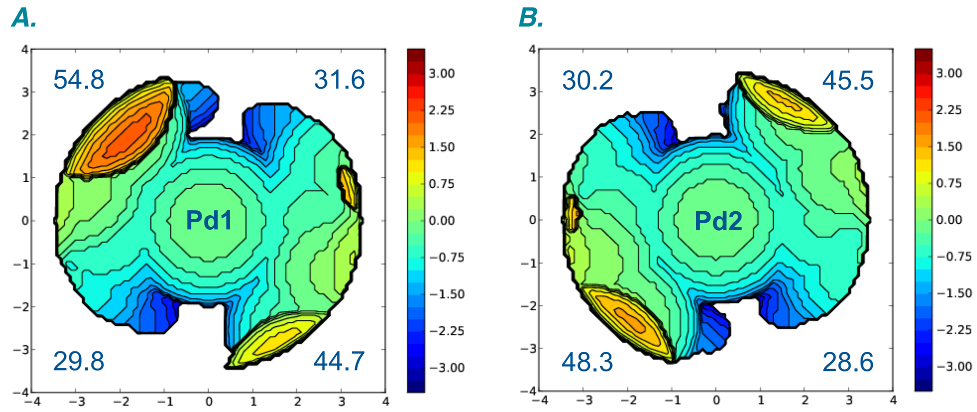

1: [IPr#-PEPPSI] (*Molecule-1*)
(*%V_{bur} = 40.2*)

1: [IPr#-PEPPSI] (*Molecule-2*)
(*%V_{bur} = 38.2*)

Table S2. *Steric Parameters of Pd–NHC Complexes Calculated from X-ray Crystallography.*

Complex	%V_{bur}	SW	NW	NE	SE
**[IPr#-PEPPSI]** (*molecule-1*)	40.2	29.8	54.8	31.6	44.7
**[IPr#-PEPPSI]** (*molecule-2*)	38.2	48.3	30.2	45.5	28.6
**[IPr-PEPPSI]**	34.8	32.7	40.7	29.1	36.7
**[IPent-PEPPSI]**	38.3	47.4	30.3	45.2	30.4
**[Pd(IPr#)(cin)Cl]**	44.7	26.9	63.7	29.9	58.2

D. Computational Methods

Computational Methods. All the calculations were performed using Gaussian 09 suite of programs. All of the geometry optimizations were performed at the B3LYP level of theory in the gas phase with the QZVP basis set for palladium and the 6-311++G(d,p) basis set for the other atoms. For geometry optimizations, we employed the X-ray structure of [IPr#–PEPPSI] as the starting geometry and performed full optimization. The absence of imaginary frequencies was used to characterize the structures as minima on the potential energy surface. All of the optimized geometries were verified as minima (no imaginary frequencies). NBO calculations were performed at the DFT/B3LYP level using NBO program implemented in Gaussian software package. Wiberg bond indices were calculated by the NBO method (*J. Mol. Struc. Theochem* **2008**, *870*, 1). Energetic parameters were calculated under standard conditions (298.15 K and 1 atm). Structural representations were generated using CYLview software (Legault, C. Y. CYL view version 1.0 BETA, University of Sherbrooke). All other representations were generated using Gauss View (GaussView, version 5, Dennington, R.; Keith, T.; Millam, J. Semichem Inc., Shawnee Mission, KS, 2009) or ChemCraft software (Andrienko, G. L. ChemCraft version b562a, https://www.chemcraftprog.com/).

Full Reference for Gaussian 09

Gaussian 09, Revision D.01, Frisch, M. J.; Trucks, G. W.; Schlegel, H. B.; Scuseria, G. E.; Robb, M. A.; Cheeseman, J. R.; Scalmani, G.; Barone, V.; Mennucci, B.; Petersson, G. A.; Nakatsuji, H.; Caricato, M.; Li, X.; Hratchian, H. P.; Izmaylov, A. F.; Bloino, J.; Zheng, G.; Sonnenberg, J. L.; Hada, M.; Ehara, M.; Toyota, K.; Fukuda, R.; Hasegawa, J.; Ishida, M.; Nakajima, T.; Honda, Y.; Kitao, O.; Nakai, H.; Vreven, T.; Montgomery, J. A., Jr.; Peralta, J. E.; Ogliaro, F.; Bearpark, M.; Heyd, J. J.; Brothers, E.; Kudin, K. N.; Staroverov, V. N.; Kobayashi, R.; Normand, J.; Raghavachari, K.; Rendell, A.; Burant, J. C.; Iyengar, S. S.; Tomasi, J.; Cossi, M.; Rega, N.; Millam, M. J.; Klene, M.; Knox, J. E.; Cross, J. B.; Bakken, V.; Adamo, C.; Jaramillo, J.; Gomperts, R.; Stratmann, R. E.; Yazyev, O.; Austin, A. J.; Cammi, R.; Pomelli, C.; Ochterski, J. W.; Martin, R. L.; Morokuma, K.; Zakrzewski, V. G.; Voth, G. A.; Salvador, P.; Dannenberg, J. J.; Dapprich, S.; Daniels, A. D.; Farkas, Ö.; Foresman, J. B.; Ortiz, J. V.; Cioslowski, J.; Fox, D. J. Gaussian, Inc., Wallingford CT, 2009.

Figure S6. *Graphical Representation of Frontier Orbitals of Complexes **[IPr#-PEPPSI]** and **[IPr-PEPPSI]** at the B3LYP 6-311++g(d,p) Level.*

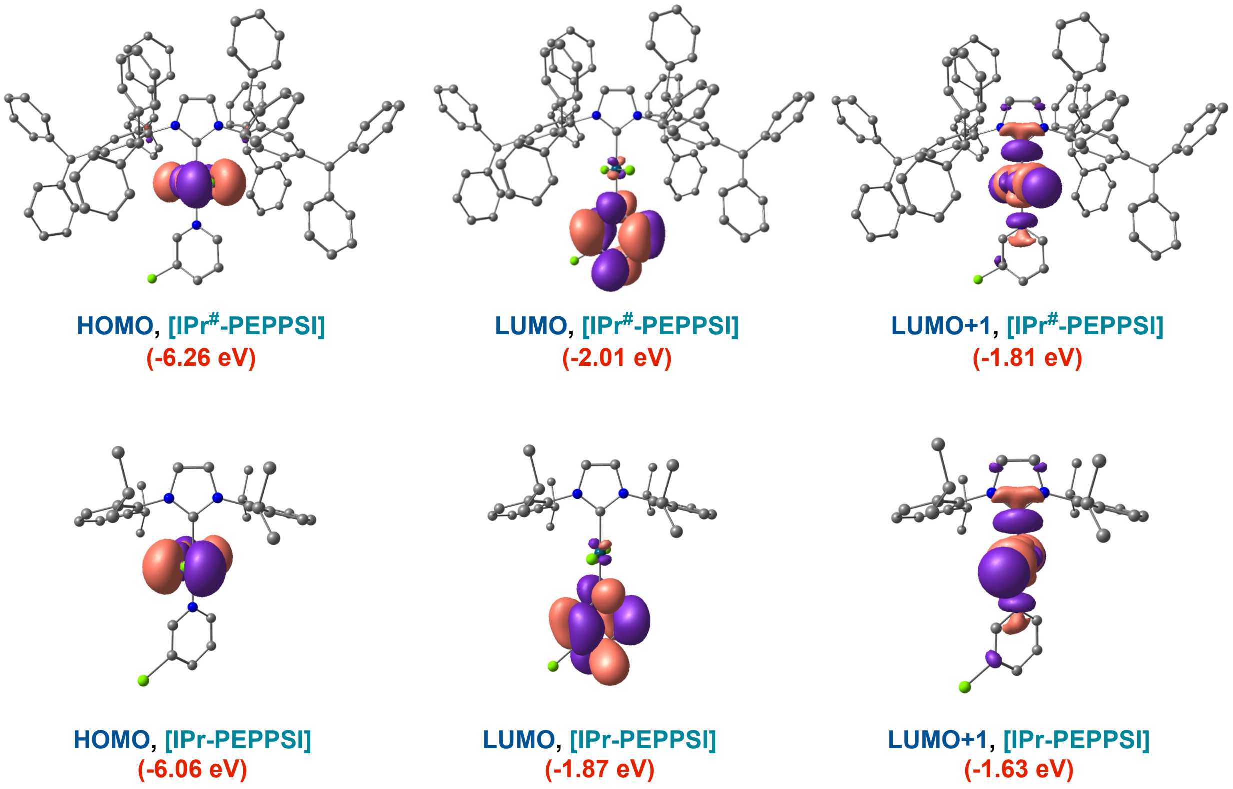

Table S3. *Frontier Orbitals and Energy of Complexes **[IPr#-PEPPSI]** and **[IPr-PEPPSI]** Calculated at the B3LYP 6-311++g(d,p) Level.*

entry	complex	orbital	E (eV)	ΔE[a] [eV]
1	**[IPr#-PEPPSI]**	HOMO	-6.26	4.25
2		LUMO	-2.01	
3		LUMO+1	-1.81	
4	**[IPr-PEPPSI]**	HOMO	-6.06	4.29
5		LUMO	-1.87	
6		LUMO+1	-1.63	

[a]$\Delta E = E_{LUMO} - E_{HOMO}$.

Figure S7. *Topographical Steric Maps of Complexes* ***[IPr#-PEPPSI]*** *and* ***[IPr-PEPPSI]*** *at the B3LYP 6-311++g(d,p) Level.*

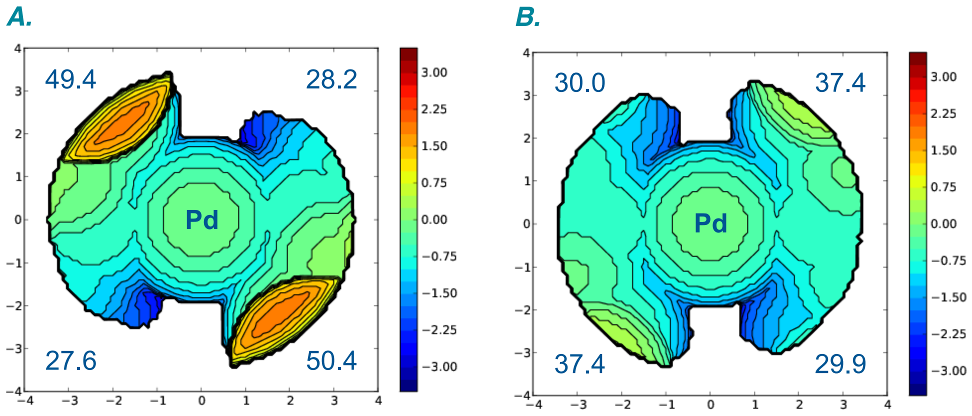

[IPr#-PEPPSI] ($\%V_{bur}$ = 38.9) **[IPr-PEPPSI]** ($\%V_{bur}$ = 33.7)

Table S4. *Steric Parameters of Complexes **[IPr$^{\#}$-PEPPSI]** and **[IPr-PEPPSI]** Calculated at the B3LYP 6-311++g(d,p) Level.*

Complex	%V_{bur}	SW	NW	NE	SE
**[IPr$^{\#}$-PEPPSI]**	38.9	27.6	49.4	28.2	50.4
**[IPr-PEPPSI]**	33.7	37.4	30.0	37.4	29.9

E. ^{1}H NMR (500 MHz, $CDCl_3$) and ^{13}C NMR (126 MHz, $CDCl_3$) of [IPr#-PEPPSI]

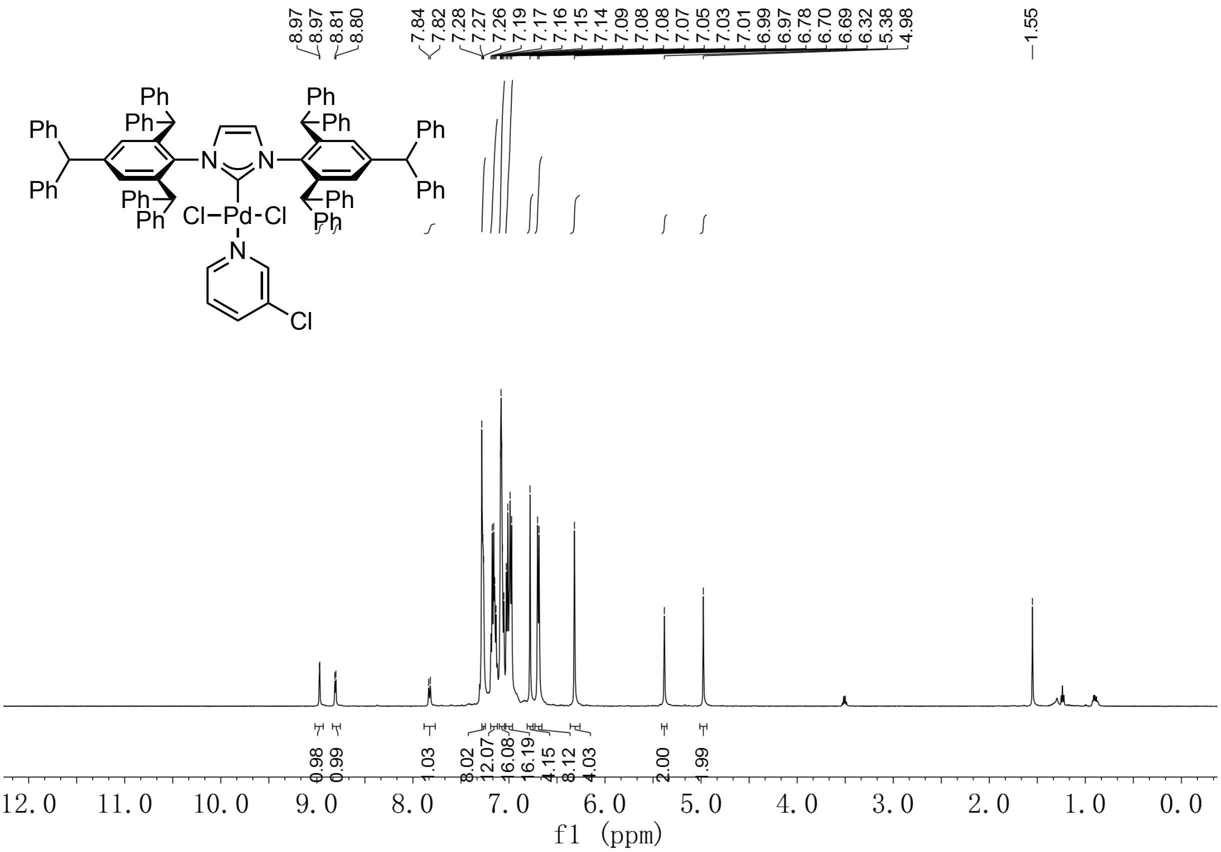

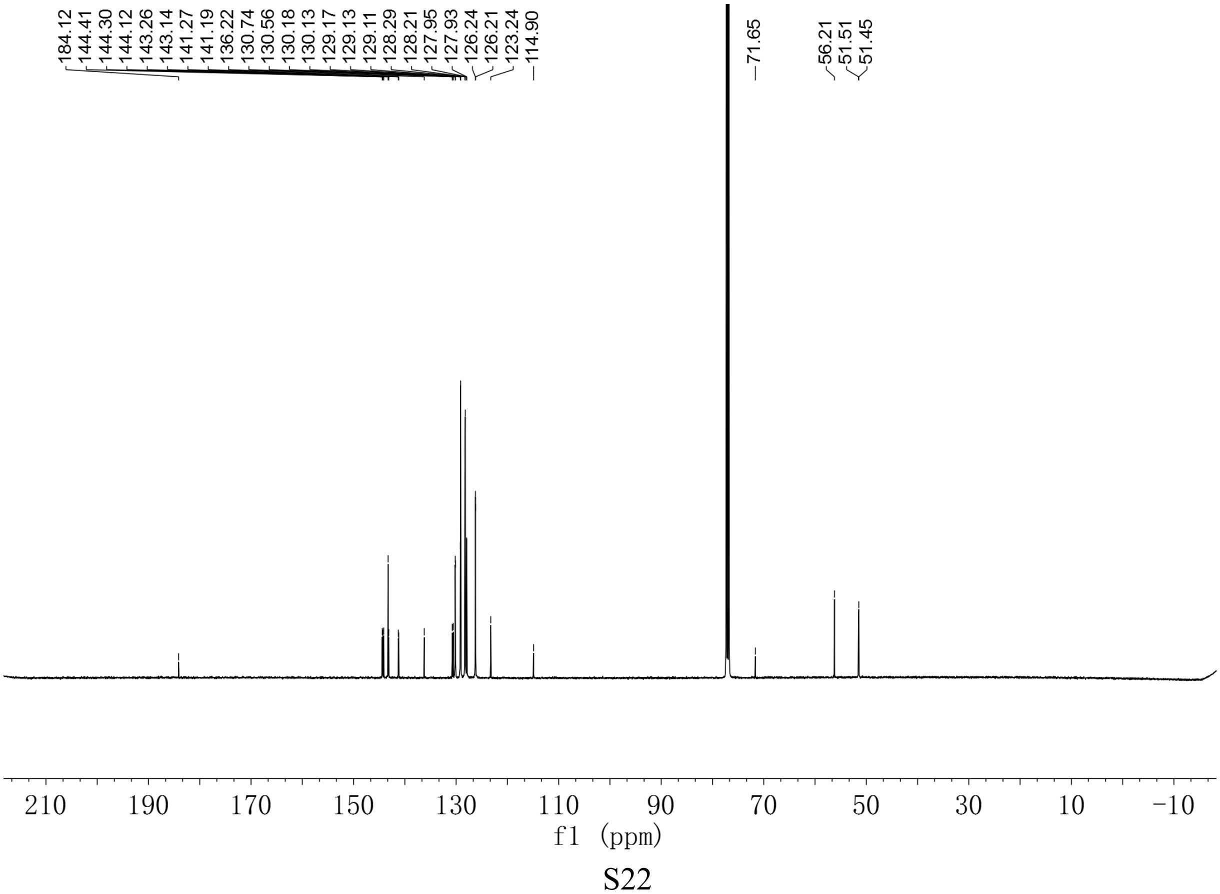

[IPr#-PEPPSI]

Energy: -5454.193243 au

Sum of electronic and thermal Energies: -5452.650417 au

Geometry:

Cl	-1.07215700	1.36314600	2.47815000
Cl	1.21231300	1.94123800	-1.56528600
Cl	-2.41543600	6.52321800	2.03259300
N	-1.02387200	-1.16847800	-0.05442000
N	1.13459100	-1.20094600	0.17349200
N	0.11916300	3.72734500	0.78401100
C	-0.61324700	-2.49247100	-0.19967800
H	-1.31049400	-3.28410500	-0.39859800
C	2.75486500	0.40438100	3.95892000
C	0.72356100	-2.51542800	-0.03806700
H	1.42004100	-3.33221900	-0.05737400
C	3.02867100	-0.37360700	1.53470800
C	3.68674300	-0.06116500	4.89360800
H	4.02352400	-1.09052100	4.85106800
C	2.52821800	-0.80663500	0.28939700
C	2.21253500	-0.48540200	2.83172800
H	1.20613300	-0.11652200	2.62502500
C	0.05574300	-0.36281900	0.17032500
C	-2.42004500	-0.76387700	-0.07757700
C	-4.23818700	0.41837200	-1.12241100
H	-4.61055800	1.03002400	-1.93320000

C	-2.91398700	-0.02747300	-1.17373800
C	3.06466900	-3.08554400	-2.11952800
C	4.18132300	0.77866100	5.89085600
H	4.90210200	0.39635800	6.60563000
C	-5.08038000	0.10929000	-0.05764400
C	4.34029500	0.10248000	1.57657400
H	4.72686600	0.47661900	2.51578300
C	5.16567300	0.11513400	0.45453600
C	-4.58295300	-0.69307400	0.96548600
H	-5.23393300	-0.95766300	1.78981800
C	-3.47468700	1.18360800	-4.36089400
H	-3.79265500	0.18006500	-4.61893200
C	-1.89651400	-1.04827600	-3.28295600
C	2.92194100	-1.55640200	-2.15911500
H	1.85793200	-1.34361200	-2.28478300
C	4.67552500	-0.42638800	-0.72979800
H	5.31788100	-0.47368700	-1.59919100
C	2.20544900	-3.86453100	-2.90512700
H	1.43587000	-3.37859400	-3.49480100
C	3.36631800	-0.89874800	-0.84291200
C	1.00842300	-2.23314600	4.19250600
H	0.31507900	-1.44910000	4.47831100
C	-3.57083200	-1.88760000	3.42905800
C	4.04148900	-3.73730200	-1.36094400

H	4.72008100	-3.16052500	-0.74380700
C	-3.26726200	-1.15466600	0.98256900
C	-2.76610500	-2.14026100	-3.22298400
H	-3.58598100	-2.13779200	-2.51447200
C	-4.68633100	-2.64676700	3.80107400
H	-5.04471300	-3.43626000	3.15171100
C	-2.92575800	-3.58574900	1.67142200
C	-2.82796700	-2.11394400	2.10048700
H	-1.77376500	-1.91373800	2.30540000
C	-5.34218500	-2.41234200	5.00983500
H	-6.20465500	-3.01395400	5.27581500
C	3.61830300	-0.97893900	-3.40354200
C	-2.07244500	0.21837800	-2.43526300
H	-1.07233800	0.51751100	-2.11573800
C	-0.84533200	-1.08851900	-4.20911200
H	-0.16547900	-0.24597800	-4.27670600
C	-2.59354500	1.38049600	-3.29192700
C	0.20159800	6.47244600	1.25045800
C	-7.51568900	-0.43486000	-0.57690600
C	2.05578200	-1.93380700	3.31069800
C	3.74512000	2.09926500	5.97643700
H	4.12410900	2.75121300	6.75571400
C	-7.23258500	-1.18149200	-1.72508400
H	-6.27400600	-1.06843700	-2.21762400

C	4.74423800	-1.57955300	-3.97773400
H	5.15043900	-2.48823000	-3.55040900
C	-3.12172200	-0.89601200	4.30910900
H	-2.26618300	-0.29148300	4.03506100
C	2.79153600	-4.24283700	3.48711100
H	3.49207600	-5.02149000	3.20517400
C	-0.97053100	4.36882300	1.23308100
H	-1.84860800	3.76881900	1.42685100
C	2.94297200	-2.95642600	2.96493500
H	3.76207800	-2.75229200	2.28530800
C	-4.88814300	-1.41967700	5.87471500
H	-5.39391900	-1.24089500	6.81717000
C	-3.94017700	2.26177900	-5.11266700
H	-4.62200200	2.08595000	-5.93772000
C	-0.95941300	5.73932300	1.46355600
C	6.56086700	0.72980500	0.55990500
H	6.83555700	0.67050300	1.61812800
C	-6.53478500	0.58060900	0.01225600
H	-6.77348700	0.63603800	1.07935900
C	-0.67395300	-2.18077900	-5.05419000
H	0.14307500	-2.18412700	-5.76757100
C	4.83586400	0.12381200	-5.69159800
H	5.30287900	0.54652900	-6.57436600
C	-6.72362100	2.00306100	-0.52735900

C	2.31701100	1.72943000	4.06123600
H	1.56968200	2.09604100	3.36784600
C	-2.17987100	2.68581700	-3.00375200
H	-1.47261200	2.85349000	-2.20030300
C	5.67276800	2.81168400	-0.67352500
H	4.88047700	2.22000200	-1.11586200
C	-2.59485600	-3.24065200	-4.06594300
H	-3.28249500	-4.07705900	-4.00147100
C	-2.64161300	3.76437000	-3.75507000
H	-2.30224000	4.76701600	-3.51859700
C	1.32502800	5.79142100	0.79546200
H	2.25725000	6.31225900	0.61771300
C	5.35043000	-1.03126800	-5.10813900
H	6.22254400	-1.51507600	-5.53443600
C	6.58417800	2.22112200	0.20629000
C	-3.52659500	3.55742300	-4.81163500
H	-3.88417700	4.39611200	-5.39868100
C	-8.16752900	-2.07623200	-2.24559900
H	-7.92788000	-2.64331600	-3.13862900
C	1.24666100	4.42417800	0.56346500
H	2.08745400	3.86225200	0.18149900
C	4.15681900	-5.12893800	-1.38589700
H	4.92347900	-5.61321900	-0.79078200
C	7.61922000	-0.08010000	-0.19536800

C	2.31594400	-5.25158500	-2.92971600
H	1.63407900	-5.83370400	-3.53998900
C	3.71022500	0.72697400	-5.13246700
H	3.29485100	1.62470500	-5.57749900
C	3.10899700	0.18022200	-4.00088300
H	2.24523700	0.66626400	-3.56455000
C	0.85800000	-3.51236100	4.71959300
H	0.04470400	-3.71743200	5.40736100
C	1.75181000	-4.52535800	4.36838100
H	1.63777400	-5.52234700	4.77931500
C	-2.05455400	-4.51760000	2.24952600
H	-1.30484300	-4.17879400	2.95593300
C	-8.75939900	-0.61525600	0.03972400
H	-8.99777700	-0.04610000	0.93278100
C	-3.77231600	-0.66357000	5.51892400
H	-3.40376400	0.11025200	6.18359300
C	2.80697300	2.56963800	5.05928800
H	2.44652500	3.59050800	5.12549400
C	3.29473400	-5.89185600	-2.16848500
H	3.38126600	-6.97253200	-2.18492200
C	-3.87517400	-4.04707900	0.75456500
H	-4.56152100	-3.34905600	0.28993700
C	7.66855600	4.38230700	0.46706700
H	8.44599200	4.98725500	0.92082400

C	-1.55128800	-3.26446400	-4.98661700
H	-1.42125000	-4.11580700	-5.64554600
C	-6.23313600	3.07438600	0.23180000
H	-5.73566000	2.87462100	1.17581700
C	-7.36074600	2.28392900	-1.73764200
H	-7.76267600	1.47568200	-2.33644600
C	8.05164300	0.25563800	-1.48110200
H	7.65758400	1.13996900	-1.96784600
C	-9.69481600	-1.51024200	-0.47441900
H	-10.65017900	-1.63545100	0.02331800
C	-9.40221600	-2.24475700	-1.62254500
H	-10.12730500	-2.94342700	-2.02460000
C	7.57898200	3.02787500	0.77412400
H	8.29403700	2.58682000	1.46167300
C	-7.49818900	3.60018500	-2.18254700
H	-7.99528100	3.79643000	-3.12622800
C	-3.95217700	-5.40184500	0.42418800
H	-4.69820600	-5.73729200	-0.28808800
C	-2.12662000	-5.86784700	1.92007900
H	-1.43561500	-6.56900500	2.37516600
C	6.76048800	4.95911200	-0.42129000
H	6.83046900	6.01321600	-0.66621200
C	8.16491500	-1.21657100	0.41487700
H	7.84570000	-1.48841200	1.41655200

C	8.99622200	-0.52944400	-2.14304300
H	9.31788000	-0.25155300	-3.14073900
C	5.76386500	4.16911000	-0.98799100
H	5.05169200	4.60331800	-1.68148300
C	-3.07835700	-6.31746400	1.00393200
H	-3.13504600	-7.36874200	0.74465600
C	9.52942600	-1.65890200	-1.52714500
H	10.26823400	-2.26461300	-2.03990100
C	-7.00534300	4.65530500	-1.42124900
H	-7.11536500	5.67740500	-1.76597300
C	9.11095800	-1.99981500	-0.24100200
H	9.52546200	-2.87184300	0.25316000
C	-6.37101800	4.38698700	-0.20767200
H	-5.98555000	5.20054100	0.39707500
H	0.22197300	7.53888000	1.43658500
Pd	0.07300900	1.61187000	0.45395400
